# Combined strategy of upfront CTCA and optimal treatment for stable chest pain: rationale and design of the CLEAR-CAD trial

**DOI:** 10.1007/s12471-024-01906-3

**Published:** 2024-10-18

**Authors:** Victor A Verpalen, Casper F Coerkamp, Mark J Hinderks, Joan G Meeder, Michiel M Winter, E Karin Arkenbout, Jeroen C Vis, Jesse Habets, Martijn W Smulders, Casper Mihl, Clara E E van Ofwegen-Hanekamp, Tycho I G van der Spoel, Wilco Tanis, Rogier E van Gelder, Marloes L J van der Wielen, G Aernout Somsen, Wouter J Kikkert, Luc F Carati, Abdelilah el Barzouhi, Paul F M M van Bergen, Admir Dedic, Mathias Prokop, Hein P Stallmann, Xavier D Y Beele, Henriëtte M E Quarles van Ufford, Robin Nijveldt, Marcel G W Dijkgraaf, Peter Damman, R Nils Planken, José P S Henriques

**Affiliations:** 1 Department of Radiology and Nuclear Medicine, Amsterdam University Medical Center, University of Amsterdam, Amsterdam Cardiovascular Sciences, Amsterdam, The Netherlands; 2 Department of Cardiology, Amsterdam University Medical Center, University of Amsterdam, Amsterdam Cardiovascular Sciences, Amsterdam, The Netherlands; 3 Department of Cardiology, Radboud University Medical Center, Nijmegen, The Netherlands; 4 Department of Cardiology, VieCuri Medical Center, Venlo, The Netherlands; 5 Cardiology Centers Netherlands (CCN), Utrecht, The Netherlands; 6 Department of Cardiology, Tergooi Hospital, Hilversum, The Netherlands; 7 Department of Cardiology, Haaglanden Medical Center, The Hague, The Netherlands; 8 Department of Radiology, Haaglanden Medical Center, The Hague, The Netherlands; 9 Department of Cardiology, Cardiovascular Research Institute Maastricht (CARIM), University Medical Center Maastricht, Maastricht, The Netherlands; 10 Department of Radiology, Cardiovascular Research Institute Maastricht (CARIM), University Medical Center Maastricht, Maastricht, The Netherlands; 11 Department of Cardiology, Diakonessenhuis, Utrecht, The Netherlands; 12 Department of Cardiology, Haga Teaching Hospital, The Hague, The Netherlands; 13 Department of Radiology, Haga Teaching Hospital, The Hague, The Netherlands; 14 Department of Cardiology, Treant Zorggroep, Scheper Hospital, Emmen, The Netherlands; 15 Department of Radiology, VieCuri Medical Center, Venlo, The Netherlands; 16 Department of Cardiology, Dijklander Hospital, Hoorn, The Netherlands; 17 Department of Cardiology, Noordwest Clinics, Alkmaar, The Netherlands; 18 Department of Radiology, Radboud University Medical Center, Nijmegen, The Netherlands; 19 Department of Radiology, Treant Zorggroep, Scheper Hospital, Emmen, The Netherlands; 20 Department of Radiology, Tergooi Hospital, Hilversum, The Netherlands; 21 Department of Epidemiology and Data Science, Amsterdam UMC, University of Amsterdam, Amsterdam, The Netherlands; 22 Methodology, Amsterdam Public Health, Amsterdam, The Netherlands

**Keywords:** Coronary artery disease, Computed tomography coronary angiography, Optimal medical therapy, Non-invasive functional imaging, Revascularisation

## Abstract

**Background:**

Patients with stable chest pain suspected of coronary artery disease (CAD) usually undergo multiple diagnostic tests to confirm or rule out obstructive CAD. Some tests may not effectively assess the presence of CAD, precluding optimal treatment. A diagnostic strategy of upfront computed tomography coronary angiography (CTCA) combined with optimal medical therapy (OMT) tailored to the extent of CAD may be superior to standard care in preventing major adverse cardiac events.

**Study design:**

The CLEAR-CAD trial is a prospective, open-label, multicentre, randomised, superiority trial of an upfront CTCA-guided strategy in 6444 patients presenting in an outpatient setting with suspected CAD compared with standard care, in approximately 30 participating centres in the Netherlands. The upfront CTCA-guided strategy consists of an initial CTCA which is assessed using the Coronary Artery Disease-Reporting and Data System (CAD-RADS 2.0). In patients without CAD (CAD-RADS 0) no specific cardiac medication is mandated. Patients with non-obstructive CAD (CAD-RADS 1–2) are treated with preventive OMT. Patients with obstructive CAD (CAD-RADS ≥ 3) are treated with preventive and anti-anginal OMT; in the presence of pharmacologically refractory symptoms patients undergo selective revascularisation after non-invasive functional imaging for myocardial ischaemia (≥ 10%). Patients with significant left main or proximal left anterior descending coronary artery stenosis on CTCA undergo direct invasive coronary angiography and subsequent revascularisation. The primary endpoint is the composite of all-cause death and myocardial infarction.

**Conclusion:**

The CLEAR-CAD trial is the first randomised study to investigate the efficacy of a combined upfront CTCA-guided medical and selective revascularisation strategy in an outpatient setting with suspected CAD compared with standard care.

**Supplementary Information:**

The online version of this article (10.1007/s12471-024-01906-3) contains supplementary material, which is available to authorized users.

## Introduction and rationale

In clinical practice, patients with stable chest pain suspected of coronary artery disease (CAD) still undergo a variety of diagnostic tests [1, 2]. These may include exercise electrocardiogram (X-ECG), (stress) echocardiography, computed tomography coronary-artery calcium score (CAC), computed tomography coronary angiography (CTCA), nuclear imaging, cardiac magnetic resonance (stress perfusion) imaging (CMR) and invasive coronary angiogram (ICA) with invasive coronary physiology or imaging. All these tests have inherent limitations, leading to potential false-positive and/or false-negative results with additional visits for downstream tests [3]. In addition, most tests do not detect non-obstructive CAD, precluding many patients from optimal primary preventive therapy. Also, many patients with positive functional testing undergoing ICA do not have obstructive CAD [4].

In the SCOT-HEART trial, the routine use of CTCA on top of standard care in the assessment of stable chest pain increased the frequency and accuracy of CAD diagnosis. The improved detection of both obstructive and non-obstructive CAD allowed targeted preventive optimal medical therapy (OMT), which is assumed to have driven the significant reduction of death from coronary heart disease and nonfatal myocardial infarction (MI) at 5‑year follow-up [5]. Currently, the European Society of Cardiology (ESC) guidelines recommend non-invasive functional imaging or CTCA as the initial diagnostic test in most patients with stable symptoms suspected of CAD [6].

The SCOT-HEART trial did not explore a CTCA-first strategy, as CTCA was added to standard care [7]. CTCA on top of standard care resulted in an increased number of total diagnostic tests in the CTCA group, leading to higher direct healthcare costs within the first 6 months [8]. Besides, there was no reduction in the number of diagnostic ICAs observed [5]. Thus far, an upfront CTCA-guided strategy for the combined diagnosis and treatment of suspected CAD has not been studied in comparison with standard care. In the DISCHARGE trial, patients with stable chest pain referred for ICA were randomised to either CTCA or ICA and showed similar major adverse cardiovascular events (MACE) rates over 3.5 years follow-up [9].

Moving from diagnosis to treatment, the ISCHEMIA and REVIVED trial showed that OMT was non-inferior to an early invasive strategy plus OMT with regard to the primary clinical outcome in patients with obstructive CAD and proven ischaemia on non-invasive imaging or exercise test without imaging [10, 11]. Therefore, ICA and revascularisation can safely be withheld in most patients and only used in a small subset of patients with pharmacological refractory symptoms.

To combine the evidence and overcome limitations of previous trials, a new randomised trial testing an upfront CTCA-guided strategy for the combined diagnosis and treatment of suspected CAD was warranted [5, 9–11]. These observations led to the design of the ‘Clinical Outcomes and Cost-effectiveness of a Diagnostic and Treatment Strategy of Upfront CTCA plus Selective Non-Invasive Functional Imaging Compared with Standard Care in Patients with Chest Pain and Suspected Coronary Artery Disease’ (CLEAR-CAD) trial. The study hypothesis is that an upfront CTCA-guided strategy combined with OMT and highly selective revascularisation, after non-invasive functional imaging for detection of myocardial ischaemia (≥ 10%) in patients with obstructive CAD and pharmacological refractory symptoms, reduces MACE.

## Methods

### Study design and population

**Fig. 1:**
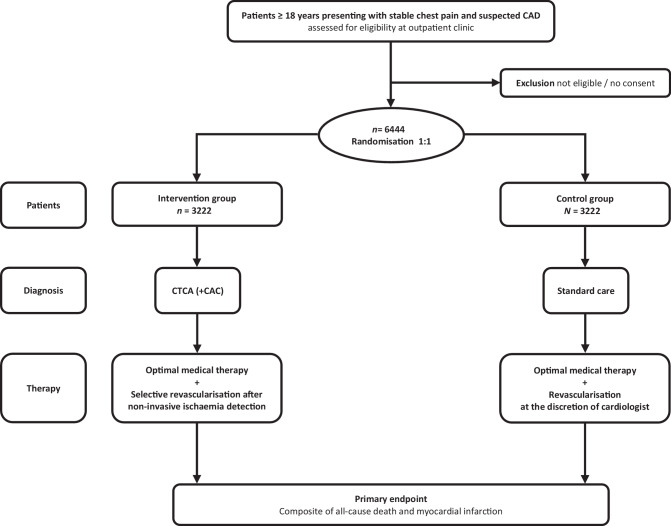
Study design (*CAC* coronary-artery calcium score, *CAD* coronary artery disease, *CTCA* computed tomography coronary angiography, *OMT* optimal medical therapy)

The CLEAR-CAD trial is a prospective, open-label, multicentre, randomised clinical trial enrolling 6444 patients referred to a cardiology outpatient clinic with stable chest pain suspected of CAD. The study specifically excludes patients with a history of CAD on cardiac imaging or those suspected of having acute coronary syndrome. The inclusion and exclusion criteria are presented in Table 1. Patients are selected for inclusion in the study at the end of the first visit to the outpatient clinic, and consenting patients are randomised in a 1:1 ratio to either the upfront CTCA-guided strategy or standard care (Fig. 1).

**Table 1:** Inclusion and exclusion criteria

Inclusion criteria	Exclusion criteria
- Outpatient presentation to the cardiologist with stable chest pain suspected for CAD and ≥ 18 years	- Presentation with or history of acute coronary syndrome (STEMI/NSTEMI/unstable angina) - History of CAD on cardiac imaging^a^ - History of PCI and/or CABG - Permanent atrial fibrillation - Severe renal failure (eGFR < 30 ml/min) - Severe allergy to iodinated contrast medium - Known pregnancy - Patients with an estimated life expectancy of less than 1 year

*CABG* coronary artery bypass grafting, *CAD* coronary artery disease, *CTCA* computed tomography coronary angiography, *eGRF* estimated glomerular filtration rate, *ICA* invasive coronary angiogram, *NSTEMI* non-ST-segment elevation myocardial infarction, *PCI* percutaneous coronary intervention and STEMI ST-segment elevation myocardial infarction

^a^History of CAD on cardiac imaging is defined as the presence of coronary calcifications on a coronary-artery calcium score, or as either non-obstructive or obstructive CAD detected by CTCA or ICA, or a positive functional test detected with dobutamine stress echocardiography, stress cardiac magnetic resonance imaging, single-photon emission computed tomography, or positron emission tomography

### Study procedures

#### Upfront CTCA-guided strategy

**Fig. 2:**
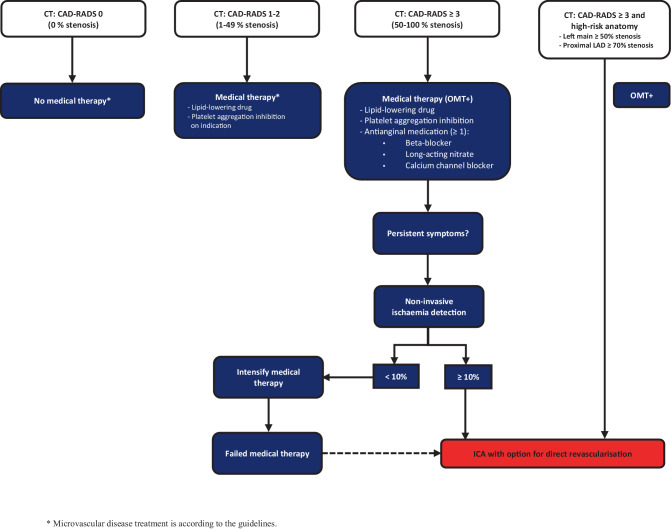
The intervention group (CTCA-guided) (*CAD-RADS* Coronary Artery Disease-Reporting and Data System, *CT* computed tomography, *ICA* invasive coronary angiography, *LAD* left anterior descending artery, *OMT* optimal medical treatment)

Patients randomised to the upfront CTCA-guided strategy (intervention) undergo CTCA (+CAC) within 6 weeks after randomisation (Fig. 2). The CTCA is assessed using the standardised Coronary Artery Disease-Reporting and Data System (CAD-RADS 2.0) method [12]. The subsequent diagnostic and treatment strategies are based on the CAD-RADS score:

- In patients with CAD-RADS 0, CAD is excluded as the cause of anginal chest pain. In these patients no specific cardiac medication is mandated.
- In patients with CAD-RADS 1–2, CTCA indicates non-obstructive CAD, excluding obstructive CAD as the cause of anginal chest pain. In these patients, preventive OMT is started, consisting of a lipid-lowering drug. Initiation of platelet aggregation inhibition is at the discretion of the treating cardiologist.
- In patients with CAD-RADS ≥ 3, CTCA indicates obstructive CAD. In these patients, the treating cardiologist starts preventive OMT consisting of both a lipid-lowering drug and a platelet aggregation inhibitor and additional anti-anginal medication (OMT+) consisting of a minimum of one of the following: beta-blocker, long-acting nitrate or calcium channel blocker. After 4–6 weeks, anginal symptoms will be evaluated by the cardiologist. In the event of persistent symptoms, additional non-invasive functional imaging will be performed within 3 months. Patients qualify for ICA in the presence of either myocardial ischaemia in at least 10% of the myocardium on nuclear perfusion via SPECT/PET or CMR stress perfusion imaging or at least 2 of 16 segments with severe hypokinesis or akinesis on stress echocardiography. In the absence of ischaemia as defined above and persisting symptoms under optimised medical therapy, ICA may be considered.
  In CAD-RADS ≥ 3 patients, a high-risk anatomy is defined as having left main (LM) diameter stenosis of at least 50% and/or a proximal left anterior descending (LAD) diameter stenosis of at least 70%. These patients do not require non-invasive ischaemia detection but are sent for direct ICA and subsequent revascularisation.
- In patients with CAD-RADS *N*, CTCA indicates a (partial) non-diagnostic examination. Management of these patients is described in Appendix C of the Electronic Supplementary Material.

Initiation of medical treatment is allowed before CTCA.

#### Standard care strategy

Patients randomised to the standard care strategy (control) are diagnosed and treated according to the current standard care as outlined in the ESC guidelines for suspected CAD [6]. In current practice, X‑ECG, CAC, CTCA, non-invasive functional imaging tests and ICA are used interchangeably [1, 2]. A slow uptake of CTCA scans in the control group is anticipated during the study [13]. The assessment of CTCA is similar in both groups. Initiation of medical treatment is allowed at any time.

### Data collection and follow-up

All data, including the initial diagnostic trajectory and treatment, are documented in the case report forms (CRF) of the web-based electronic data capture system Castor. Follow-up is performed by telephone at 1, 3, 6 and 12 month(s) and every other year until follow-up at 5 years has been completed in all patients. In the event of the suspected occurrence of clinical endpoints, full source clinical data are collected from the hospital and general practitioners.

Assessment of symptoms of angina, dyspnoea and quality of life are assessed by the Seattle Angina Questionnaire (SAQ), the Rose Dyspnea Scale, and the EuroQol-5D (EQ-5D-5L) at the corresponding follow-up time points. Patients are also asked to complete the Institute for Medical Technology Assessment (iMTA), Medical Consumption Questionnaire (iMCQ) and the Productivity Cost Questionnaire (iPCQ) at 3, 6 and 12 months and every other year until follow-up at 5 years has been completed in all patients. All questionnaires are sent by email or post.

### Study endpoints and statistical analysis

#### Primary endpoint

The primary endpoint is the composite of all-cause death and MI according to the fourth Universal definition [14]. The main analyses of the primary endpoint are based on the time from randomisation to the first occurrence of any component of the primary endpoint. These analyses are performed in the full analysis population of all randomised patients under application of the intention-to-treat principle, which means events are counted irrespective of their occurrence relative to implementation of the randomised strategy (upfront CTCA or control). Kaplan-Meier estimates of cumulative risk and cumulative hazard functions are provided to evaluate the timing of event occurrence in the different treatment groups and the consistency of the respective treatment effects for all time points. The hazard ratio (HR), of upfront CTCA versus standard care, and corresponding two-sided 95% confidence intervals (CIs) are estimated based on Cox proportional hazards models.

The study was designed to test the following hypotheses in this hierarchical order to preserve type 1 error rate; (i) the upfront CTCA-guided strategy is non-inferior to standard care in terms of the primary endpoint, (ii) the upfront CTCA-guided strategy is superior to standard care in terms of the primary endpoint.

Non-inferiority of the upfront CTCA regimen (vis-à-vis standard care) in terms of the primary endpoint is declared if the 95% CI of the HR excludes 1.62. Superiority of the upfront CTCA regimen (vis-à-vis standard care) in terms of the primary endpoint is declared if the 95% CI of the HR excludes 1, which is equivalent to *p* < 0.05 for the log-rank test. Use of 95% CI is equivalent to non-inferiority testing with a one-sided type I error (*α*) of 0.025 and to superiority testing with a two-sided type I error rate of 0.05.

Based on the literature, the cumulative incidences at 3 years were estimated at 3.0% (control) and at 1.75% (intervention) respectively [5]. Because of an anticipated greater use of CTCA up to 30% in the control group, we estimated that a 3-year event rate in the control group would be equal to 2.82%. Assuming an exponential distribution, a target relative risk at 3 years of 0.6206 (a reduction of the 3‑year cumulative incidence from 2.82 to 1.75%) corresponds to a HR of 0.617. With 5900 fully evaluable patients and a median duration of follow-up of 3 years, the study is designed with 80% power to demonstrate the superiority of the upfront CTCA-guided strategy over standard care. The study is event-driven and the primary analysis will be performed when the number of 135 events has been reached. Follow-up is censored at the last date of known outcome status for the occurrence of death or myocardial infarction or at the global end-of-study date, which is defined as the day of occurrence of the 135 events. To account for an expected rate of lost to follow-up of 5–7%, this study will enrol 6444 patients. With a total of 135 primary endpoints, the study has a 80% power to show non-inferiority of the upfront CTCA-guided strategy to standard care, using a non-inferiority margin of 1.62 for the HR. The non-inferiority margin of 1.62 is approximately equal to the inverse of the expected therapeutic benefit (1.0/0.617).

#### Ranked secondary endpoints

If the incidence of the primary endpoint is significantly lower in the upfront CTCA group than in the control group (*p* < 0.05), then the ranked secondary endpoints are tested for superiority of CTCA over standard care in a hierarchical manner at a significance level of 0.05 to preserve the alpha level (Table 2). The time-to-event analysis methods are similar to those described for the primary endpoint. In these analyses, testing for superiority is not preceded by testing for non-inferiority. An overview of additional endpoints and endpoint definitions, is shown in Appendix B of the Electronic Supplementary Material.

**Table 2:** Study endpoints

*Primary endpoint*
Time until the occurrence of:
– Primary outcome is the composite of all-cause death and myocardial infarction
*Ranked secondary endpoints*^*a*^
Time until the occurrence of:
– All-cause death, myocardial infarction or stroke
– Cardiovascular death or myocardial infarction
– All-cause death
– Cardiovascular death
– Myocardial infarction
*Other endpoints*^*b*^
*Primary health economic outcome*
– Primary health economic outcome is defined as the costs per QALY

*QALY* quality-adjusted life-year

^a^The ranked secondary endpoints are tested in hierarchical order after testing of the primary endpoint to preserve type I error rate

^b^Other endpoints are described in Appendix B of the Electronic Supplementary Material

#### Subgroup analysis

The statistical analysis of the subgroups will be documented in the statistical analysis plan. Baseline characteristics that define subgroups of interest include age, sex, pre-test probability according to the Diamond and Forrester score [15], pre-test likelihood based on the Risk Factor-weighted Clinical Likelihood (RF-CL) model [16], cardiovascular disease risk prediction according to the cardiovascular risk using the SIGN guidelines (ASSIGN) score [17], and the Duke Clinical Score [18], angina symptom severity, blood cholesterol level and patient comorbidities such as diabetes, hypertension and renal failure. Subgroups defined by imaging characteristics include the CAC score, CAD-RADS score, coronary lesion location and characteristics, reduced left ventricular function, and ischaemia severity. Subgroups defined by follow-up results include OMT therapy adherence and lifestyle.

#### Cost-effectiveness analysis

The primary cost-effectiveness outcome is defined as the costs per quality-adjusted life-year (QALY). The economic evaluation of the upfront CTCA-guided strategy in patients with stable chest pain and suspected CAD will be accomplished as a cost-utility analysis from a societal perspective with the costs per QALY as primary outcome. In addition, cost-effectiveness analyses with the costs per MACE and costs per year progression-free of MACE will be performed to optimise the guidelines and treatment. The time horizon exceeds 12 months, so health effects and costs beyond the first year will be (differentially) discounted. A more detailed Health Economic Analysis Plan will be written prior to the start of the economic analyses [19].

### Computed tomography coronary angiography

CTCA (+CAC) will be performed using at least a 64-multidetector scanner in all patients. The minimal standards and protocol guidance have been described in the standard operating procedures, which is outlined in Appendix C of the Electronic Supplementary Material. This includes the most important patient aspects (patient information, preparation, instruction), scanner aspects (specific scan—and reconstruction protocols as well as contrast injection protocols), and reporting aspects (interpretation and reporting standards). The standards and scan protocols of all included sites will be assessed and feedback will be provided before start of inclusion for overall high quality scan acquisition, interpretation and reporting. Enrolment will only start after meeting the prescribed minimal standards.

### Study oversight and funding

The study was designed in accordance with the principles of the Declaration of Helsinki.

The CLEAR-CAD trial is registered on ClinicalTrials.gov with the unique identifier NCT05344612 (www.clearcad.nl). The study is designed and sponsored by the Amsterdam University Medical Center and Radboud University Medical Center. The Steering Committee is responsible for the study design, trial execution, data analysis and reporting of results. All primary and secondary endpoints are adjudicated by an independent Clinical Event Committee (CEC), blinded to the assigned study group. An independent Data and Safety Monitoring Board (DSMB) provides external oversight to ensure the safety of the study patients. The CLEAR-CAD trial is funded by a research grant from the Care Evaluation and Appropriate Use (‘Zorgevaluatie en Gepast Gebruik’) program of Dutch Organisation for Health Research and Development (ZonMw).

**Fig. 3:**
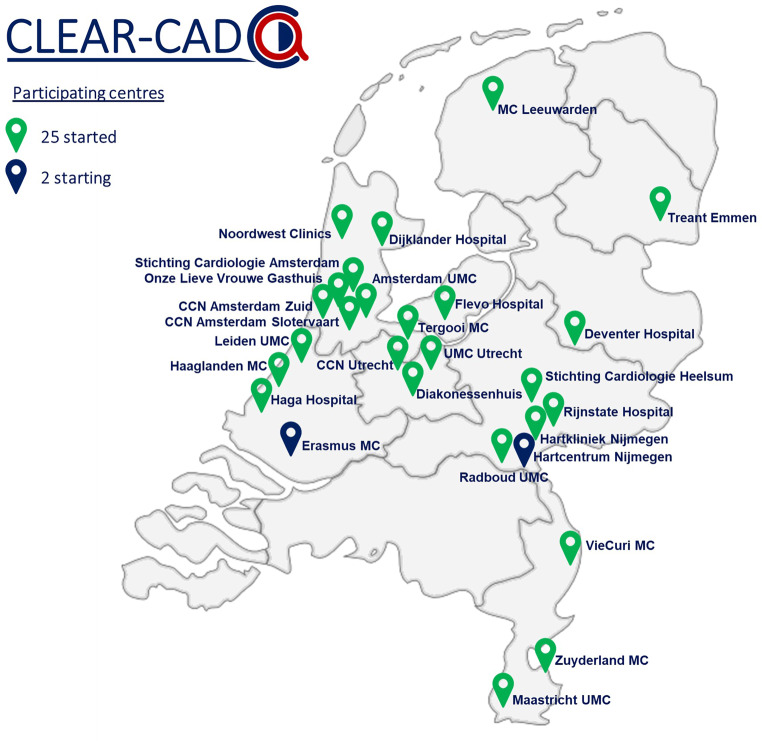
CLEAR-CAD participating centres (*CCN* Cardiology Center Netherlands*, MC* medical centre, *UMC* university medical centre)

The first patient was randomised on 14 September 2022 and complete enrolment is expected by the end of 2025. At present, 25 sites (of the current 27 participant sites) have started enrolment and randomised approximately 3300 patients (Fig. 3). The main results of the trial are expected to be available by the end of 2027.

## Discussion

The multicentre, randomised, clinical CLEAR-CAD trial aims to evaluate an upfront CTCA-guided strategy in 6444 patients presenting with stable chest pain suspected of CAD. The upfront CTCA-guided strategy is combined with optimal medical therapy and highly selective revascularisation after non-invasive functional imaging for detection of myocardial ischaemia in patients with obstructive CAD and pharmacological refractory symptoms. This study aims to demonstrate superiority of the upfront CTCA-guided strategy on the composite primary endpoint of all-cause death and MI compared with standard care.

It is common knowledge that CTCA is able to accurately rule out CAD with an overall sensitivity of 95%, rendering it an excellent first-line test for patients with stable chest pain suspected of CAD [20]. Landmark CTCA trials have demonstrated that the use of CTCA in these patients resulted in higher diagnostic yield for detecting CAD [7, 9, 21]. The early diagnosis of both obstructive and non-obstructive CAD allows to guide and intensify preventive medical therapy, thereby potentially reducing future cardiovascular events [5].

CLEAR-CAD is unique as none of the previous CTCA trials investigated a combined upfront CTCA-guided medical and selective revascularisation strategy compared with standard care (Table 3 for the landmark CTCA trials [5, 7, 9, 21] and Table S1 of the Electronic Supplementary Material for all the randomised CTCA trials [5, 7, 9, 21–32]).

**Table 3:** Landmark randomised CTCA trials in patients with stable chest pain suspected of CAD

Study, (year)	*N*	Design		Known CAD excluded	Patients (PTP)	Standardised CTCA reporting	Prespecified medical strategy in CTCA group	Prespecified invasive strategy in CTCA group	Primary endpoint	Results
		Intervention	Control							
PROMISE, (2015)	10003	CTCA	Functional imaging	No^a^	Low to intermediate, 53.4 ± 21.4 vs 53.2 ± 21.4^b^	No	No	No	All-cause death, MI, hospitalisation for unstable angina, or complications (FUP median 25 months)	3.3% vs 3.0% *p* = 0.75
SCOT-HEART, (2015)	4146	Standard care +CTCA (+CAC)	Standard care	No^c^	Low to intermediate, 18 ± 11 vs 17 ± 12^d^	No	No	No	Certainty of angina diagnosis caused by CAD at 6 weeks	HR 1.79 (1.62–1.96); *p* < 0.0001
SCOT-HEART, (2018)^e^									Death from CAD or MI (FUP 5 years)	2.3% vs 3.9% *p* = 0.004
DISCHARGE, (2022)	3561	CTCA (+CAC)	ICA	Yes	Intermediate, 36.6 (28.8–46.2) vs 37.9 (29.5–46.5)^f^	No	OMT indicated: ≥ 1 VD ≥ 20%	ICA indicated: High risk anatomy (LM ≥ 50%, proximal LAD ≥ 50% or 3 VD ≥ 50%) *OR* 1–2 VD ≥ 50% and ≥ 10% ischaemia	Cardiovascular death, MI, stroke (FUP median 3.5 years)	2.1% vs 3.0% *p* = 0.10
CLEAR-CAD, (2027)	6444	CTCA (+CAC)	Standard care	Yes	Unselected	Yes (CAD-RADS)	OMT indicated: CAD-RADS ≥ 1 *AND* OMT + indicated: CAD-RADS ≥ 3	ICA indicated: High risk anatomy (LM ≥ 50% or proximal LAD ≥ 70%) *OR* CAD-RADS ≥ 3 with persistent symptoms and ≥ 10% ischaemia	All-cause death or MI (event driven)	NA

*Vessel %* diameter stenosis coronary artery, *CAC* coronary artery calcium score, *CAD* coronary artery disease, *CAD-RADS* coronary artery disease-reporting and data system, *CTCA* computed tomography coronary angiography, *FUP* follow-up, *HR* hazard ratio, *ICA* invasive coronary angiography, *LAD* left anterior descending artery, *LM* left main, *MI* myocardial infarction, *NA* not available, *OMT* preventive optimal medical therapy, *OMT+* preventive combined with anti-anginal optimal medical therapy, *PTP* pre-test probability, *VD* vessel disease

^a^CAD exclusion criteria: known CAD with prior MI, PCI, CABG or any angiographic evidence of CAD ≥ 50% lesion in a major epicardial vessel

^b^Combined Diamond and Forrester score and Coronary Artery Surgery Study risk scores range from 0 to 100, with higher scores indicating a greater likelihood of obstructive CAD

^c^CAD exclusion criteria: acute coronary syndrome within 3 months

^d^10 years coronary heart disease risk prediction according to the cardiovascular risk using SIGN guidelines (ASSIGN) score

^e^5 year follow-up of SCOT-HEART trial

^f^Pre-test probability of obstructive CAD in patients with chest pain according to the Diamond and Forrester score

Also, in previous trials, the use of CTCA led to a higher ICA use [21, 33], and this is especially important given the low rate of ICA leading to revascularisation after CTCA [34]. In the CLEAR-CAD trial, ICA and potential revascularisation will be reserved for patients with pharmacological refractory symptoms and substantial ischaemia (≥10%) or high-risk anatomy defined as LM ≥ 50% diameter stenosis or LAD ≥ 70% diameter stenosis.

In addition to better clinical outcomes, a cost saving is also expected with the CLEAR-CAD strategy. The Dutch national healthcare costs for CAD amounted to 2.4 billion euros in 2015 and are expected to increase to 4.2 billion euros in 2030 [1]. The CLEAR-CAD upfront CTCA-guided strategy may result in yearly cost savings of millions of euros, considering a yearly national patient population of 180,000 with suspected CAD [1].

In conclusion, the CLEAR-CAD trial is the first randomised study investigating an upfront CTCA-guided strategy combined with optimal medical therapy and highly selective revascularisation after non-invasive functional imaging for detection of myocardial ischaemia (≥ 10%) in stable patients suspected of CAD compared with standard care. The findings from CLEAR-CAD are expected to significantly impact clinical practice and guideline recommendations, ultimately leading to improved clinical outcomes, patient care and lower healthcare costs.

## Supplementary Information

Supplementary Appendix
