# Correction to: Combined strategy of upfront CTCA and optimal treatment for stable chest pain: rationale and design of the CLEAR-CAD trial

**DOI:** 10.1007/s12471-026-02071-5

**Published:** 2026-08-12

**Authors:** Victor A. Verpalen, Casper F. Coerkamp, Mark J. Hinderks, Joan G. Meeder, Michiel M. Winter, E. Karin Arkenbout, Jeroen C. Vis, Jesse Habets, Martijn W. Smulders, Casper Mihl, Clara E. E. van Ofwegen-Hanekamp, Tycho I. G. van der Spoel, Wilco Tanis, Rogier E. van Gelder, Marloes L. J. van der Wielen, G. Aernout Somsen, Wouter J. Kikkert, Luc F. Carati, Abdelilah el Barzouhi, Paul F. M. M. van Bergen, Admir Dedic, Mathias Prokop, Hein P. Stallmann, Xavier D. Y. Beele, Henriëtte M. E. Quarles van Ufford, Robin Nijveldt, Marcel G. W. Dijkgraaf, Peter Damman, R. Nils Planken, José P. S. Henriques, CLEAR-CAD investigators

**Affiliations:** 1 Department of Radiology and Nuclear Medicine, Amsterdam University Medical Center, University of Amsterdam, Amsterdam Cardiovascular Sciences, Amsterdam, The Netherlands; 2 Department of Cardiology, Amsterdam University Medical Center, University of Amsterdam, Amsterdam Cardiovascular Sciences, Amsterdam, The Netherlands; 3 Department of Cardiology, Radboud University Medical Center, Nijmegen, The Netherlands; 4 Department of Cardiology, VieCuri Medical Center, Venlo, The Netherlands; 5 Cardiology Centers Netherlands (CCN), Utrecht, The Netherlands; 6 Department of Cardiology, Tergooi Hospital, Hilversum, The Netherlands; 7 Department of Cardiology, Haaglanden Medical Center, The Hague, The Netherlands; 8 Department of Radiology, Haaglanden Medical Center, The Hague, The Netherlands; 9 Department of Cardiology, Cardiovascular Research Institute Maastricht (CARIM), University Medical Center Maastricht, Maastricht, The Netherlands; 10 Department of Radiology, Cardiovascular Research Institute Maastricht (CARIM), University Medical Center Maastricht, Maastricht, The Netherlands; 11 Department of Cardiology, Diakonessenhuis, Utrecht, The Netherlands; 12 Department of Cardiology, Haga Teaching Hospital, The Hague, The Netherlands; 13 Department of Radiology, Haga Teaching Hospital, The Hague, The Netherlands; 14 Department of Cardiology, Treant Zorggroep, Scheper Hospital, Emmen, The Netherlands; 15 Department of Radiology, VieCuri Medical Center, Venlo, The Netherlands; 16 Department of Cardiology, Dijklander Hospital, Hoorn, The Netherlands; 17 Department of Cardiology, Noordwest Clinics, Alkmaar, The Netherlands; 18 Department of Radiology, Radboud University Medical Center, Nijmegen, The Netherlands; 19 Department of Radiology, Treant Zorggroep, Scheper Hospital, Emmen, The Netherlands; 20 Department of Radiology, Tergooi Hospital, Hilversum, The Netherlands; 21 Department of Epidemiology and Data Science, Amsterdam UMC, University of Amsterdam, Amsterdam, The Netherlands; 22 Methodology, Amsterdam Public Health, Amsterdam, The Netherlands

**Correction to:**

**Neth Heart J 2024**

10.1007/s12471-024-01906-3

In the article titled “Combined strategy of upfront CTCA and optimal treatment for stable chest pain: rationale and design of the CLEAR-CAD trial”, there was an error in table 2 in the Ranked secondary endpoints section. The list of ranked secondary endpoints should have also included ‘Myocardial Infarction’ as the last and separate endpoint.

This erratum corrects the ‘Ranked secondary endpoints’ section in accordance with the original study protocol. The original article has been corrected.

