# Supplementary material for: Combined strategy of upfront CTCA and optimal treatment for stable chest pain: rationale and design of the CLEAR-CAD trial
Source: Neth Heart J. 2024 Oct 18;32(11):387–96. doi: 10.1007/s12471-024-01906-3 (PMC11502636; doi:10.1007/s12471-024-01906-3)
Supplement: Supplementary file 1 — Supplementary Appendix [file 12471_2024_1906_MOESM1_ESM.docx]

**Supplemental material**

**Table S1.** All randomised CTCA trials in patients with stable chest pain suspected of CAD

| **First author/ study, (year)** | **N** | **Design** | | **Known CAD excluded** | **Patients (PTP)** | **Standardized**  **CTCA reporting** | **Prespecified medical strategy in CTCA group** | **Prespecified invasive strategy  in CTCA group** | **Primary Endpoint** | **Results** |
| --- | --- | --- | --- | --- | --- | --- | --- | --- | --- | --- |
|  |  | **Intervention** | **Control** |  |  |  |  |  |  |  |
| Min et al, (2012) | 180 | CTCA | SPECT | Yes | Low, intermediate and high,  18.3 ± 12.7 vs 19.2 ± 13.7* | No | No | No | Near-term angina-specific health status at an average of 55 days | Both arms improved comparably |
| PROMISE, (2015) | 10003 | CTCA | Functional imaging | No | Low to intermediate, 53.4 ± 21.4 vs 53.2 ± 21.4† | No | No | No | All-cause death, MI, hospitalisation for unstable angina, or major procedural complications within 72 hours (FUP median 25 months) | 3.3% vs 3.0%; P = 0.75 |
| SCOT-HEART, (2015)  SCOT-HEART, (2018)§ | 4146 | Standard care + CTCA (+CAC) | Standard care | No | Low to intermediate, 18 ± 11 vs  17 ± 12‡ | No | No | No | Certainty of angina diagnosis caused by CAD at 6 weeks  Death from CAD or MI (FUP 5 years) | HR 1.79 (1.62-1.96); P < 0.0001  2.3% vs 3.9%; P = 0.004 |
| CAPP,  (2015) | 500 | CTCA (+CAC) | Exercise stress ECG | Yes | Low, intermediate, and high, 44.9 ± 30.2 vs 47.8 ± 31.7¶ | No | No | No | The change of the SAQ score at 3 months | Angina stability:  -11.1 (-17.4 to -4.8: CTCA better); P = 0.001; Quality of life: -5.7 (-10.3 to -1.2: CTCA better);  P = 0.014; Other SAQ domains were similar. |
| CRESCENT I, (2016) | 350 (2:1) | CAC (+CTCA) | Functional imaging | Yes | Intermediate,  45 ± 29 vs  45 ± 29¶ | No | No | No | Absence of chest pain complaints at 1 year | 39% vs 25%; P = 0.012 |
| CAD-Man, (2016) | 340 | CTCA (+CAC) | ICA | No | Intermediate, 31.3 ± 21.1 vs 37.3 ± 24.8‖ | No | Any non-calcified coronary plaque: statin treatment  Only calcified coronary plaque: no statin recommended | Revascularisation indicated: obstructive CAD on CTCA and ≥50% myocardial viability on late enhancement MRI | Major procedural (CTCA or ICA) complication occurring within 48 hours | 0.6% vs 0.0%; P = 1.00 |
| IAEA-SPECT/CTCA, (2017) | 303 | CTCA (+CAC) | SPECT | Yes | Intermediate*¶ | No | No | No | Additional non-invasive testing or ICA within 6 months | 27.7% vs 16.8%; P = 0.023 |
| CRESCENT II,  (2018) | 268 | CAC (+CTCA +CTP) | Functional imaging | No | Intermediate to high, 56 ± 30 vs 53 ± 30¶ | No | No | ICA indicated: obstructive CAD on CTCA and ≥10% ischemia on CTP | Rate of ICA without a ESC class 1 indication for revascularisation at 6 months | 1.5% vs 7.2%; P = 0.035 |
| CAT-CAD, (2018) | 120 | CTCA | ICA | No | Intermediate to high,  90.0% vs 86.7%  High, 10.0% vs 13.3%¶ | No | No | No | Rate of ICA within 3 months  ICA not leading to revascularisation within 3 months | 35% vs 98%; P < 0.001  8% vs 70%; P < 0.001 |
| CONSERVE, (2019) | 1611 | CTCA | ICA | Yes | Intermediate, 51 ± 30 vs  52 ± 30¶ | No | No | No | All-cause death, MI, unstable angina, stroke, urgent and/or emergent revascularisation or cardiac hospitalisation (FUP 1 year) | 4.6% vs 4.6%; P = 0.99 |
| CARE-CCTA, (2019) | 903 | CTCA | SPECT | No | Intermediate, 43.5 ± 23.6 vs 45.3 ± 23.4¶ | No | No | No | Total cost  All-cause death, ACS, cerebrovascular accident, revascularisation, stent thrombosis, or significant bleeding (FUP 1 year) | $4,514 vs $5,208; P = 0.043  4.6% vs 5.4%; P = 0.455 |
| RESCUE, (2020) | 1050 | CTCA | SPECT | No | Unknown | No | OMT indicated: ≥1 VD ≥50% | ICA indicated: LM ≥50% **OR** ≥1 VD ≥50% without LM disease with persistent angina despite OMT | Cardiac death, MI, or revascularisation  (FUP median 16.2 months) | HR 1.03 (0.61-1.75); P = 0.19 |
| Reis et al, (2022) | 220 | CTCA (+CAC) | ICA | No | Low to Intermediate, 55.8 ± 29.2 vs 58.6 ± 25.1‖ | No | No | No | Diagnostic yield of ICA (detection of obstructive CAD) | 84.4% vs 41.9%; P < 0.001 |
| DISCHARGE, (2022) | 3561 | CTCA (+CAC) | ICA | Yes | Intermediate, 36.6 (28.8-46.2) vs 37.9 (29.5-46.5)¶ | No | OMT indicated: ≥1 VD ≥20% | ICA indicated:  High risk anatomy  (LM ≥50%, proximal LAD ≥50% or 3 VD ≥50%) or N in high risk anatomy **OR**  1-2 VD ≥50% or N in 1-2 vessels without high risk anatomy and ≥10% ischemia | Cardiovascular death, MI, stroke (FUP median 3.5 years) | 2.1% vs 3.0%; P = 0.10 |
| CLEAR-CAD, (2027) | 6444 | CTCA (+CAC) | Standard care | Yes | Unselected | Yes  (CAD-RADS) | OMT indicated:  CAD-RADS ≥1  **AND**  OMT+ indicated: CAD-RADS ≥3 | ICA indicated:  High risk anatomy  (LM ≥50% or proximal LAD ≥70%) **OR**  CAD-RADS ≥3 with persistent symptoms despite OMT+ and ≥10% ischemia | All-cause death or MI (event driven) | NA |

Vessel % denotes diameter stenosis coronary artery, ACS acute coronary syndrome, CAC coronary artery calcium score, CAD coronary artery disease, CAD-RADS coronary artery disease-reporting and data system, CTCA computed tomography coronary angiography, CTP computed tomography perfusion, ESC european society of cardiology, FUP follow-up, HR hazard ratio, ICA invasive coronary angiography, LAD left anterior descending artery, LM left main, LVEF left ventricular ejection fraction, MI myocardial infarction, MRI magnetic resonance imaging, NA not available, OMT preventive optimal medical therapy, OMT+ preventive combined with anti-anginal optimal medical therapy, OR odds ratio, PTP pretest probability, SAQ seattle angina questionnaire, SPECT singel-photon emission computed tomography, VD vessel disease.
* 10-year risk of manifesting clinical cardiovascular disease according to the Framingham risk score in asymptomatic patients.
† Combined Diamond and Forrester score and Coronary Artery Surgery Study risk scores range from 0 to 100, with higher scores indicating a greater likelihood of obstructive CAD.
‡ Cardiovascular disease risk prediction according to the cardiovascular risk using SIGN guidelines (ASSIGN) score.
§ 5 year follow-up of SCOT-HEART trial.
¶ Pretest probability of obstructive CAD in patients with chest pain according to the Diamond and Forrester score.
‖ Pretest probability of obstructive CAD in patients with chest pain according to the Duke clinical score.

**Supplement to: V.A. Verpalen et al: CLEAR-CAD rationale and design**

**Contents:**

**Appendix A**

1. CLEAR-CAD study organisation.……………………………………………......….. 4
2. Site lead investigators………………………………………………………………... 6
3. CLEAR-CAD consortium investigators……………………………………………... 9

**Appendix B**

1. All endpoints………………………………………………………………………….17
2. Endpoint definitions…………………………………………………………………..18

**Appendix C**

1. CTCA SOP and protocol……………………………………………………………...24
2. Non-invasive functional imaging modalities SOP and protocols……………………..32

**Appendix A**

1. **CLEAR-CAD study organisation**

**Chair**

Prof. J.P.S. Henriques

**Co-chair**

Dr. R.N. Planken

Dr. P. Damman

**Steering Committee**

Prof. J.P.S. Henriques, Dr. R.N. Planken, Dr. P. Damman, Prof. J.B. van Goudoever, Prof. M.G.W. Dijkgraaf, Dhr. W. Dubbeldam, Prof. R. Nijveldt, Prof. R. Vliegenthart, Dr. H.J Verberne, Prof. P. van der Harst, Prof. R.P.J. Budde, Dr. J. Habets, Dr. J.G. Meeder

**Extended Committee**

Prof. M. Prokop, Dr. H.M.E. Quarles van Ufford, Dr. S.M. Boekholdt, Prof. N. Van Royen, Dr. A.H.G. Driessen, Prof. T. Leiner, Prof. H.M. Den Ruiter, Dr. S. Elias, Dr. M. Voskuil, Dr. K. Zwinderman, Dr. M.M. Winter, Prof. P. Knaapen, Dr. M.F.L. Meijs, Dr. A. Hirsch, Prof. I. Isgum, Dr. S. El Messaoudi, Prof. H.J. Lamb, Dr. V.A.W.M. Umans, Dr. J. Hjortnaes, Prof. R.H.J.A. Slart, Dr. L.M. De Heer, Prof. J.W. Jukema

**Chair cardiology**

Prof. J.P.S. Henriques

Dr. P. Damman

**Chair radiology**

Dr. R.N. Planken

**Chair CMR imaging**

Prof. R. Nijveldt

**Chair Nuclear medicine**

Dr. H.J. Verberne

**Chair cardiothoracic surgery**

Dr. I.J. Wijdh-den Hamer

**Chair Health-Economic Outcome**

Prof. M.G.W. Dijkgraaf

M.S van Barreveld

**Independent Expert**

Dr. L.F.H.J Robbers

**Statistical and Data Coordinating Centre (SDCC)**

Amsterdam University Medical Center

**Data safety and monitor board (DSMB)**

Prof. J.G.P. Tijssen (chair)

Prof. F.W.A. Verheugt (member)

Dr. M.F. Boomsma (member)

**Clinical Event Committee (CEC)**

Dr. L. Timmers (cardiologist)

Dr. P.A.L. Tonino (cardiologist)

1. **Site lead investigators (cardiologist, radiologist)**

Amsterdam Universitair Medisch Centrum

Prof. J.P.S. Henriques

Dr. R.N. Planken

Cardiologie Centra Nederland Amsterdam Slotervaart

Dr. M.M. Winter

Dr. R.N. Planken

Cardiologie Centra Nederland Amsterdam Zuid

Dr. G.A. Somsen

Dr. R.N. Planken

Cardiologie Centra Nederland Amsterdam Utrecht

Prof. L. Hofstra

Dr. R.N. Planken

Deventer Ziekenhuis

Dr. A. van der Sluis

Dr. I.D. Kilsdonk

Diakonessenhuis

Dr. C.E.E. van Ofwegen-Hanekamp

Drs. D.B. Naafs

Dijklander Ziekenhuis

Drs. L. van Oosterom

Dr. A. Slaar

Flevoziekenhuis

Dr. R. Franken

Dr. J.H.D. de Bruine

Haaglanden Medisch Centrum

Dr. J.C. Vis

Dr. J. Habets

Haga Ziekenhuis

Dr. W. Tanis

Dr. R.E. van Gelder

Hartcentrum Nijmegen

Dr. M.A. Brouwer

Dr. K. Salah

Hartkliniek Nijmegen

Dr. P.C. Kievit

Dr. K. Salah

Leids Universitair Medisch Centrum

Dr. P.R.M. van Dijkman

Prof. H.J. Lamb

Maastricht Universitair Medisch Centrum

Dr. M.W. Smulders

Dr. C. Mihl

Medisch Centrum Leeuwarden

Dr. P. Woudstra

Dr. A.H.A. Mazairac

Noordwest Ziekenhuisgroep

Dr. A. Dedic

Dr. R.J.J. Knol

Onze Lieve Vrouwe Gasthuis

Dr. M.A. Vink

Dr. A. Driessen-Waaijer

Radboud Universitair Medisch Centrum

Dr. P. Damman

Dr. K. Salah

Rijnstate

Dr. R. Pisters

Drs. M.L.E. Bernsen

Stichting Cardiologie Amsterdam

Dr. J.B. Winter

Dr. R.N. Planken

Stichting Cardiologie Heelsum

Dr. F.A.M. Jonkman

Dr. K. Salah

Tergooi Ziekenhuizen

Dr. E.K. Arkenbout

Dr. X.D.Y. Beele

Treant

Dr. M.L.J. van der Wielen

Dr. H.P. Stallmann

Universitair Medisch Centrum Utrecht

Prof. P. van der Harst

Prof. B.K. Velthuis

VieCuri Medisch Centrum

Dr. J.G. Meeder

Drs. L.F. Carati

Zuyderland

Dr. M. Versteylen

Dr. G.K. Wong

1. **CLEAR-CAD consortium investigators**

Cardiologie Centra Nederland Amsterdam Zuid

G.A. Somsen (cardiologist)

I.I. Tulevski (cardiologist)

F.R. den Hartog (cardiologist)

H.W. Vliegen (cardiologist)

J.J. Koolen (cardiologist)

H.A. Hauer (cardiologist)

L.H.B. Baur (cardiologist)

M. Overhagen (cardiologist)

H. Sen (cardiologist)

R.N. Planken (radiologist)

O.M. Mets (radiologist)

A. van Randen (radiologist)

J. van Schuppen (radiologist)

A.M. Spijkerboer (radiologist)

R. Sprengers (radiologist)

R.A.P. Takx (radiologist)

L. Meijboom (radiologist)

L. Brunekreeft (radiologist)

K.J. Franssen (physician assistant radiology)

N.H.J. Lobé (CTCA technician)

I. Kroon (nurse)

C. Schneider (nurse)

A. Schiele (nurse)

Cardiologie Centra Nederland Amsterdam Slotervaart

M.M. Winter (cardiologist)

J.S. Lemkes (cardiologist)

A. Nap (cardiologist)

F.J. de Lange (cardiologist)

M. El Amrani (cardiologist)

R.S.S. Kort (cardiologist)

K. Miedema (cardiologist)

J.W. Buikema (cardiologist)

C.J.W. Verouden (cardiologist)

V.D. van der Zwan (cardiologist)

F.W.A. Verheugt (cardiologist)

R.N. Planken (radiologist)

O.M. Mets (radiologist)

A. van Randen (radiologist)

J. van Schuppen (radiologist)

A.M. Spijkerboer (radiologist)

R. Sprengers (radiologist)

R.A.P. Takx (radiologist)

L. Meijboom (radiologist)

L. Brunekreeft (radiologist)

K.J. Franssen (physician assistant radiology)

N.H.J. Lobé (CTCA technician)

H. de Kruif (nurse)

E. de Beukelaer (nurse)

I. Kroon (nurse)

M. Meijer (nurse)

K. Wenzel (nurse)

Tergooi Ziekenhuizen

E.K. Arkenbout (cardiologist)

W.J. Kikkert (cardiologist)

R.J. van Bommel (cardiologist)

X.D.Y. Beele (radiologist)

E. Verduyn (research nurse)

G. Nendels (research assistant)

Haaglanden Medisch Centrum

J.C. Vis (cardiologist)

A. El Barzouhi (cardiologist)

M.S. Buiten (cardiologist)

E.G.M. Olde Bijvank (cardiologist)

S.F.A. Askar (cardiologist)

K.A. Kortekaas (cardiologist)

A. Dereci (cardiologist)

E. Schrage (cardiologist)

H.J.A. Hensing (physician assistant cardiology)

J. Habets (radiologist)

S.A. Rebergen (radiologist)

D.W. Kaandorp (radiologist)

A. Verschuuren-Koers (CTCA technician)

P. Sorensen (research nurse)

G. Algoe-Ramasre (research assistant)

VieCuri Medisch Centrum

J.G. Meeder (cardiologist)

S. Heijmans (cardiologist)

G. Tjeerdsma (cardiologist)

W.S. Remkes (cardiologist)

S. Aydin (cardiologist)

F. Eerens (cardiologist)

L.F. Carati (radiologist)

C.W.K.P. Arnoldussen (radiologist)

T. Yu (radiologist)

I. al Younis (nuclear medicine physician)

J. Sanders-Verkoelen (research nurse)

W. van Elsbergen (research nurse)

Diakonessenhuis

C.E.E. van Ofwegen-Hanekamp (cardiologist)

T.I.G. van der Spoel (cardiologist)

A.M. Wind (cardiologist)

M. Wiertsema (cardiologist)

J.G.J. Groothuis (cardiologist)

H.J. Houtgraaf (cardiologist)

M. Epping (research fellow cardiology)

D.B. Naafs (radiologist)

M. Bontje (research nurse)

N. Plazier (research nurse)

M. de Jong (research nurse)

Haga Ziekenhuis

W. Tanis (cardiologist)

M. Bax (cardiologist)

P.W.A. Janssen (cardiologist)

F. Nijhoff (cardiologist)

V. Murdoch (physician assistant cardiology)

R.E. van Gelder (radiologist)

C.J. van Rooden (radiologist)

N. de Roode (research nurse)

A. van den Heuvel (research nurse)

Maastricht Universitair Medisch Centrum

M.W. Smulders (cardiologist)

A.W.J. van ’t Hof (cardiologist)

K. Hendriks (cardiologist)

S.A.F. Streukens (cardiologist)

J.F.A. Ubachs (cardiologist)

P.A. Vriesendorp (cardiologist)

G.P. Bijvoet (cardiologist)

A. Lux (cardiologist)

K. Vernooy (head Department of Cardiology)

C. Mihl (radiologist)

B. Martens (radiologist)

J.E. Wildberger (head Department of Radiology and Nuclear Medicine)

M.P.H. Jacobs - Dols (research nurse)

M.A.H. Baggen (research nurse)

Treant

M.L.J. van der Wielen (cardiologist)

L. Kleijn (cardiologist)

H.P. Stallmann (radiologist)

A.C. van de Werp (radiologist)

D.B. Meek (radiologist)

W.A.S. Slijkhuis (radiologist)

L. Schaafsma (research nurse)

H. Smit (research nurse)

Y. Nieuwhof (research nurse)

Dijklander Ziekenhuis

P.F.M.M. van Bergen (cardiologist)

E. Wierda (cardiologist)

L. Oosterom (cardiologist)

A. Slaar (radiologist)

C.P.M. van Nes (research nurse)

Deventer Ziekenhuis

A. van der Sluis (cardiologist)

E.A. Badings (research doctor cardiology)

I.D. Kilsdonk (radiologist)

B. Hazenbos (research nurse)

L. Ebels (research nurse)

W. Tousain (research nurse)

Radboud Universitair Medisch Centrum

P. Damman (cardiologist)

A.C. Leen (cardiologist)

J.L. Bonnes (cardiologist)

S. El Messaoudi (cardiologist)

G.A. de Waard (cardiologist)

L.X. van Nunen (cardiologist)

M.M.P. Driesen (cardiologist)

P.F.A. Teunissen (resident cardiology)

D.H.F. Gommans (resident cardiology)

S.A.J. Damen (resident cardiology)

M.E.C.J. Hassel (resident cardiology)

S.P.G. van Vught (resident cardiology)

G.C. Swart (resident cardiology)

L.M.J. Wijnands (resident cardiology)

A. Yaksh (resident cardiology)

M.J. Hinderks (research fellow cardiology)

K. Salah (radiologist)

M. Prokop (radiologist)

M. Brink (radiologist)

M. Snoeren (radiologist)

M. Huisman (radiologist)

H. Schalkx (radiologist)

Noordwest Ziekenhuisgroep

A. Dedic (cardiologist)

V.A.W.M. Umans (cardiologist)

C.E.P. Siegers (cardiologist)

R.J.J. Knol (nuclear medicine physician)

J. Last (research nurse)

H. Bakke (research nurse)

P. Mol (research nurse)

S. van Lieshout (research nurse)

L. Boon (research nurse)

Onze Lieve Vrouwe Gasthuis

M.A. Vink (cardiologist)

N. Colman (cardiologist)

R.G.E.J. Groutars (cardiologist)

R. Halbmeijer (cardiologist)

M.L. Lieuw-A-Fa (cardiologist)

A. Driessen-Waaijer (radiologist)

A. Boersma (research fellow cardiology)

E.L. Schade (research fellow cardiology)

B. van der Wouden (research nurse)

Rijnstate

R. Pisters (cardiologist)

Y. America (cardiologist)

M.L.E. Bernsen (radiologist)

M. van Werkum (radiologist)

K. Meinen-Werner (research nurse)

B. Baten (research nurse)

F. Peerlings-Terki (research nurse)

Stichting Cardiologie Amsterdam

J.B. Winter (cardiologist)

M.G.C. Pieterse (cardiologist)

R.N. Planken (radiologist)

Z.M. Bijl (research assistant)

F.E. Beerman (research assistant)

Stichting Cardiologie Heelsum

F. Demirel (cardiologist)

O. Klerks (cardiologist)

K. Salah (radiologist)

M. Prokop (radiologist)

M. Brink (radiologist)

M. Snoeren (radiologist)

M. Huisman (radiologist)

H. Schalkx (radiologist)

Hartkliniek Nijmegen

E. Oudejans (cardiologist)

S. Altuntas (cardiologist)

K. Salah (radiologist)

M. Prokop (radiologist)

M. Brink (radiologist)

M. Snoeren (radiologist)

M. Huisman (radiologist)

H. Schalkx (radiologist)

Leids Universitair Medisch Centrum

P.R.M. van Dijkman (cardiologist)

M.V. Regeer (cardiologist)

J.M.J. Booger (cardiologist)

H.J. Lamb (radiologist)

S. Bijl (research nurse)

E. van der Willik (research nurse)

Universitair Medisch Centrum Utrecht

P. van der Harst (cardiologist)

J.W. Balder (resident cardiology)

B.K. Velthuis (radiologist)

Amsterdam Universitair Medisch Centrum

J.P.S. Henriques (cardiologist)

M. Hollander (resident cardiology)

W.J. Stuijfzand (resident cardiology)

C.F. Coerkamp (research fellow cardiology)

R.N. Planken (radiologist)

O.M. Mets (radiologist)

A. van Randen (radiologist)

J. van Schuppen (radiologist)

A.M. Spijkerboer (radiologist)

R. Sprengers (radiologist)

R.A.P. Takx (radiologist)

L. Meijboom (radiologist)

L. Brunekreeft (radiologist)

V.A. Verpalen (research fellow Radiology and Nuclear Medicine)

K.J. Franssen (physician assistant radiology)

N.H.J. Lobé (CTCA technician)

E.A. Aarsman (research nurse)

Medisch Centrum Leeuwarden

P. Woudstra (cardiologist)

A.H.A. Mazairac (radiologist)

M. van der Wal (head research cardiology)

Cardiologie Centra Nederland Utrecht

Prof. L. Hofstra (cardiologist)

Dr. R.N. Planken (radiologist)

Flevoziekenhuis

Dr. R. Franken (cardiologist)

Dr. J.H.D. de Bruine (radiologist)

Hartcentrum Nijmegen

Dr. M.A. Brouwer (cardiologist)

Dr. K. Salah (radiologist)

Zuyderland

Dr. M. Versteylen (cardiologist)

Dr. S. Rasoul (cardiologist)

Dr. G.K. Wong (radiologist)

**Appendix B**

1. **All endpoints**

**Primary endpoint**

The primary endpoint is the composite of all-cause death and myocardial infarction.

**Ranked secondary endpoints**
The following secondary endpoints are tested in hierarchical order after testing of the primary endpoint to preserve type I error rate.

- Death and myocardial infarction and stroke
- Cardiovascular death and myocardial infarction
- All-cause death
- Cardiovascular death

**Other endpoints**

- Minor Cardiovascular Events (MICE): They include coronary revascularisation, peripheral artery revascularisation, sudden cardiac arrest, hospitalisation for chest pain/discomfort, emergency department visit for chest pain/discomfort, transient ischemic attack, bleeding (BARC ≥2) and congestive heart failure.
- Diagnostic procedures (non-invasive and invasive)
- Invasive procedures and revascularisations
- Complications of diagnostic and therapeutic procedures
- Incidental findings on diagnostics
- Percentage and composition of OMT
- Indicators of successful OMT, including blood pressure and blood cholesterol level
- Radiation exposure
- Angina (SAQ-7 angina questionnaire)
- Dyspnea (Rose dyspnea scale)
- Quality of life (EQ-5D)

**Health economic evaluation outcomes**

The primary health economic outcome is the costs per QALY.
Secondary outcomes include:

- Volumes and costs of resources used
- Health utility
- Costs per MACE
- Costs per year progression-free of MACE

1. **Endpoint definitions**

**Death**All deaths are considered cardiac unless an alternate cause is unequivocally established even among patients with serious non-cardiac comorbidities. Death is classified according to the Academic Research Consortium definition as outlined in Supplementary Table 1.

**Supplementary Table 1. Classification of death**

| **Type** | **Definition** |
| --- | --- |
| Cardiovascular death | Death resulting from cardiovascular causes. The following categories may be collected:   - Death caused by acute myocardial infarction - Death caused by sudden cardiac, including unwitnessed, death - Death resulting from heart failure - Death caused by stroke - Death caused by cardiovascular procedures - Death resulting from cardiovascular hemorrhage - Death resulting from other cardiovascular cause |
| Non-cardiovascular death | Death that is not thought to be the result of a cardiovascular cause. The following categories may be collected:   - Death resulting from malignancy - Death resulting from pulmonary causes - Death caused by infection (includes sepsis) - Death resulting from gastrointestinal causes - Death resulting from accident/trauma - Death caused by other non-cardiovascular organ failure - Death resulting from other non-cardiovascular cause |
| Undetermined | Undetermined cause of death is defined as a death not attributable to any other category because of the absence of any relevant source documents. Such deaths will be classified as cardiovascular for endpoint determination. |

**Myocardial infarction**Myocardial infarction is defined according to the 4^th^ universal definition of myocardial infarction as outlined in Supplementary Table 2.

**Supplementary Table 2. Classification of myocardial infarction**

| **Type** | **Definition** |
| --- | --- |
| Myocardial infarction  (type 1, 2 and 3) | The term acute myocardial infarction should be used when there is acute myocardial injury with clinical evidence of acute myocardial ischemia and with detection of a rise and/or fall of cTn values with at least one value above the 99th percentile URL and at least one of the following:   - Symptoms of myocardial ischemia; - New ischemic ECG changes; - Development of pathological Q waves; - Imaging evidence of new loss of viable myocardium or new regional wall motion abnormality in a pattern consistent with an ischemic etiology; - Identification of a coronary thrombus by angiography or autopsy (not for type 2 or 3). - Post-mortem demonstration of acute athero-thrombosis in the artery supplying the infarcted myocardium meets criteria for type 1 MI. - Evidence of an imbalance between myocardial oxygen supply and demand unrelated to acute athero-thrombosis meets criteria for type 2 MI. - Cardiac death in patients with symptoms suggestive of myocardial ischemia and presumed new ischemic ECG changes before cTn values become available or abnormal meets criteria for type 3 MI. |
| Procedure-related  myocardial infarction  (types 4 and 5) | Percutaneous coronary intervention (PCI) related MI is termed type 4a MI. Coronary artery bypass grafting (CABG) related MI is termed type 5 MI. Coronary procedure-related MI #48 hours after the index procedure is arbitrarily defined by an elevation of cTn values >5 times for type 4a MI and >10 times for type 5 MI of the 99th percentile URL in patients with normal baseline values. Patients with elevated pre-procedural cTn values, in whom the preprocedural cTn level are stable (≤20% variation) or falling, must meet the criteria for a >5 or >10 fold increase and manifest a change from the baseline value of >20%. In addition with at least one of the following:   - New ischemic ECG changes (this criterion is related to type 4a MI only). - Development of new pathological Q waves. - Imaging evidence of loss of viable myocardium - Angiographic findings consistent with a procedural flow-limiting complication such as coronary dissection, occlusion of a major epicardial artery or graft, side-branch occlusion-thrombus, disruption of collateral flow or distal embolization. - Isolated development of new pathological Q waves meets the type 4a MI or type 5 MI criteria with either revascularisation procedure if cTn values are elevated and rising. - Other types of 4 MI include type 4b MI stent thrombosis and type 4c MI restenosis that both meet type 1 MI criteria. - Post-mortem demonstration of a procedure-related thrombus meets the type 4a MI criteria or type 4b MI criteria if associated with a stent. |
| Prior or silent  myocardial infarction | Any one of the following criteria meets the diagnosis for prior or silent/ unrecognized MI:   - Abnormal Q waves with or without symptoms in the absence of non-ischemic causes. - Imaging evidence of loss of viable myocardium in a pattern consistent with ischemic etiology. - Patho-anatomical findings of a prior MI. |

**Stroke**Stroke is classified as outlined in Supplementary Table 3.

**Supplementary Table 3. Classification of Stroke**

| **Type** | **Definition** |
| --- | --- |
| Stroke | An acute symptomatic episode of neurological dysfunction, more than 24 hours in duration in the absence of therapeutic intervention or death, due to cerebral, spinal or retinal tissue injury as evidenced by neuroimaging or lumbar puncture. It includes the following sub-classifications: |
| Ischemic stroke | Infarction due to prolonged ischemia. Causes include (but are not limited to) arterial and venous thrombosis, embolism, and systemic hypo perfusion |
| Hemorrhagic stroke | Caused by a non-traumatic intraparenchymal, intraventricular or subarachnoid hemorrhage |
| Undetermined stroke | Stroke with insufficient information to determine ischemic or hemorrhagic cause |
| Transient ischemic attack | Transient episode of neurological dysfunction (<24 hours) caused by temporary cerebral, spinal or retinal ischemia with no evidence of acute infarction on neuroimaging |

**Bleeding**Bleeding will be classified according to the Bleeding Academic Research Consortium criteria as outlined in Supplementary Table 6. Only relevant bleeding (BARC ≥2) will be recorded in the study.

**Supplementary Table 4. Classification of bleeding**

| **Type** | **Definition** |
| --- | --- |
| 0 | No evidence of bleeding |
| 1 | Bleeding that is not actionable and does not cause the patient to seek unscheduled performance of studies, hospitalization, or treatment by a health-care professional; may include episodes leading to self-discontinuation of medical therapy by the patient without consulting a health-care professional |
| 2 | Any overt, actionable sign of hemorrhage (e.g., more bleeding than would be expected for a clinical circumstance, including bleeding found by imaging alone) that does not fit the criteria for type 3, 4, or 5 but does meet at least one of the following criteria:   - requiring nonsurgical, medical intervention by a health-care professional, - leading to hospitalization or increased level of care, or - prompting evaluation |
| 3 | Clinical, laboratory and/or imaging evidence of bleeding with specific healthcare provider responses as listed below: |
| 3a | - Overt bleeding plus hemoglobin drop of 3 to < 5 g/dL* (provided hemoglobin drop is related to bleed) - Any transfusion with overt bleeding |
| 3b | - Overt bleeding plus hemoglobin drop ≥5 g/dL* (provided hemoglobin drop is related to bleed), - Cardiac tamponade, - Bleeding requiring surgical intervention for control (excluding dental/nasal/skin/hemorrhoid), - Bleeding requiring intravenous vasoactive agents |
| 3c | - Intracranial hemorrhage (does not include microbleeds or hemorrhagic transformation, does include intraspinal) |
| 4 | CABG-related bleeding   - Perioperative intracranial bleeding within 48 h, - Reoperation after closure of sternotomy for the purpose of controlling bleeding - Transfusion of ≥ 5 U whole blood or packed red blood cells within a 48-h period, - Chest tube output more than or equal to 2L within a 24-h period |
| 5 | Fatal bleeding |
| 5a | - Probable fatal bleeding; no autopsy or imaging confirmation but clinically suspicious |
| 5b | - Definite fatal bleeding; overt bleeding or autopsy or imaging confirmation |

**Complication Coronary Angiography (+/- Percutaneous Coronary Intervention)**Complications of coronary angiography include the following complications:

• None

• Death

• Non-fatal myocardial infarction

• Coronary/aorta dissection

• Cardiogenic shock

• Tamponade

• Retroperitoneal bleeding

• VT or VF

• Cardiac arrest

• Contrast allergy

• Contrast nephropathy

• Other complication leading to hospital admission > 24h

• Complication due to puncture site (bleeding, occlusion, dissection, (pseudo) aneurysm)

**Complication Computed Tomography Coronary Angiography (CTCA)**Complications of computed tomography coronary angiography include the following complications:

• None

• Contrast medium extravasation

• Contrast allergy

• Contrast nephropathy

**Sudden cardiac arrest**
Sudden cardiac arrest is the sudden cessation of cardiac activity such that the victim becomes unresponsive, with either persisting gasping respirations or absence of any respiratory movements, and no signs of circulation as manifest by the absence of a perceptible pulse. An arrest is presumed to be of cardiac etiology unless it is known or likely to have been caused by trauma, drowning, respiratory failure or asphyxia, electrocution, drug overdose, or any other non-cardiac cause (20).

**Admission for heart failure**Hospitalization or Emergency Department visit for heart failure is defined as below. Hospitalization is defined as an admission to an inpatient unit (following discharge after the index hospitalization) or a visit to an emergency department after randomisation that results in at least a 12 hour stay (or a date change if the time of admission/discharge is not available). Only hospitalizations that occur on an emergency (unplanned) basis will be considered as potential events. If competing causes of hospitalization or ED visits (i.e. CV and non-CV) judged to be of equal importance are at hand, the CV cause should take preference.

There must be:

1. Clinical manifestations of worsening heart failure including at least one of the following:

- New or worsening: dyspnea, orthopnea, paroxysmal nocturnal dyspnea, edema, pulmonary basilar crackles, jugular venous distension, worsening renal function with no other apparent cause or radiological evidence of worsening heart failure.

2. Additional/increased therapy specifically for the treatment of worsening heart failure with at least one of the following:

- Intravenous treatment with diuretic, inotrope, vasodilator or other recognized intravenous heart failure treatment, or
- Mechanical or surgical intervention (mechanical circulatory support, heart transplantation or ventricular pacing to improve cardiac function,) or the use of ultrafiltration, hemofiltration or dialysis that is specifically directed at treatment of heart failure.

**Appendix C:**

**I. CTCA SOP and protocols**

**CLEAR-CAD step by step CTCA approach**

**1.**     **Patient preparation (according to SKMS recommendations)**

- Full explanation of the exam
- Heart rate lowering medication (recommended dosing scheme)

o   B-blocker days before CTCA (metoprolol retard) if high (>55 bpm*) or irregular heart rate

o   B-blocker hours before CTCA exam (metoprolol tartrate) if high or irregular heart rate*

- HR 55-60 bpm → 25 mg
- HR 60-65 bpm → 50 mg
- HR 66-80 bpm → 100 mg
- HR >80 bpm → 150 mg

* if systolic blood pressure >100 mmHg, no severe aortic valve stenosis, no second/third grade AV block and no phosphodiesterase type 5 inhibitor drug (sildenafil, Viagra)

- 12-24 hour prior to CTCA NO caffeine
- Venous access (18G recommended) right antecubital vein (pressure limit 300 psi)
- Patient positioning AP scanogram
  - Offset the patient to the right so the heart is at the center of the scan field
- Place arms above their head with ECG leads outside scan range
- Administer sublingual nitrates (0,4-0,8 mg), just before CTCA

**2.**     **Breath-hold training**

- Practice breath-holding, should be the same as during scan (recorded voice instruction)
- Single “breath in and hold” command, if possible with an extra pre delay (advice 6 seconds) after breathing instruction for optimize lowering cardiac heart rate.
- Comfortable breath in
  - To avoid Valsalva maneuver and to avoid substantial heart rate fluctuation

**3.**     **Calcium scoring scan**

- Cover the range from trachea bifurcation to the apex of the heart
- Field of View (FOV) 200 mm

**4.**     **Determine start-end position CTCA scan**

- LAD often located more superior than LM (use the CAC-scan for optimal planning)
- Advice (1cm extra margin both superior and inferior if inconsistent breath-hold)

**5.**     **Select CTCA scanner settings**

- Prospective adaptive sequential scan mode, (high-pitch spiral not preferable)
- Select lowest tube voltage selection (if available) if tube current does not reach the maximum

**6.**     **Contrast injection protocol**

- Select the appropriate injection protocol based on CTCA kilovoltage (kV) and body weight (see appendix CLEAR-CAD-IDR)
  - It is recommended that the iodine delivery rate (IDR) is adjusted to a specific combination of body weight and kV

**7.**     **CTCA scan contrast timing**

- Use test-bolus (preferred) or bolus-tracking to determine the scan delay
- Region of interest (ROI) for test-bolus in ascending aorta (to determine the time-to-peak in seconds), inject contrast (10 ml recommended) followed by NaCl chaser (40 ml recommended)
- ROI for bolus-tracking: descending aorta (apply a trigger threshold >180 hounsfield units (HU) recommended). Inject the full contrast bolus followed by NaCl chaser (40 ml recommended)
- Scan delay (coronary filling time)
  - Test-bolus: 4 seconds (recommended)
  - Bolus-tracking: 2 seconds (recommended)

**8.**     **CTCA contrast injection protocol**

- Select the applicable IDR for patient body weight and selected kV from the IDR table in the appendix

**9.**     **Final check before start CTCA scan**

- Reassure patient position, comfort, breathing instruction
- Inform patient that during the next breath-hold is the most important and last scan
- Confirm that heart rate is steady and compatible with scanning protocol
- Start CTCA scan

**10.**  **Reconstruction recommendations**

- CAC settings according to vendor specific standard CAC protocol
- CTCA true-stack, <0.9 mm slice thickness, 0.25-0.4 increment, smooth kernel, filtered backprojection (FBP)
- CTCA true-stack, <0.9 mm slice thickness, 0.25-0.4 increment, sharp kernel, iterative reconstruction (moderate level)
- CTCA 1 mm slice thickness, 1 mm increment, maximum FOV for assessment of incidental findings

**11.**  **Reporting**

Protocol

- Scan mode
- Dose-length product (DLP) and CT dose index volume (CTDIvol)
- Contrast medium (brand, generic, concentration) and applied injection protocol
- Heart rate during cardiology outpatient visit, during CTCA presentation and during CTCA acquisition
- Medication (days prior to – and during the day of CTCA exam)

Image quality

- Scan-range (entire coronary tree)
- Coronary attenuation (325 – 500 HU))
- Artifacts (motion, blooming, step, etc.)

Calcium score

- Per coronary artery, total and segment involvement score (SIS) (see appendix plaque burden score)

CTCA

- CAD-RADS (see appendix CAD-RADS)

Incidental findings

**Coronary artery segments**

Main branch

- RCA: 3 segments* from aorta to PDA, proximal (1), mid (2), distal (3)
- LM: 1 segment (5) **
- LAD: 3 segments***, proximal (6), mid (7), distal (8)
- CX: 2 segments****, proximal (11), distal (13)

Side branches

- **
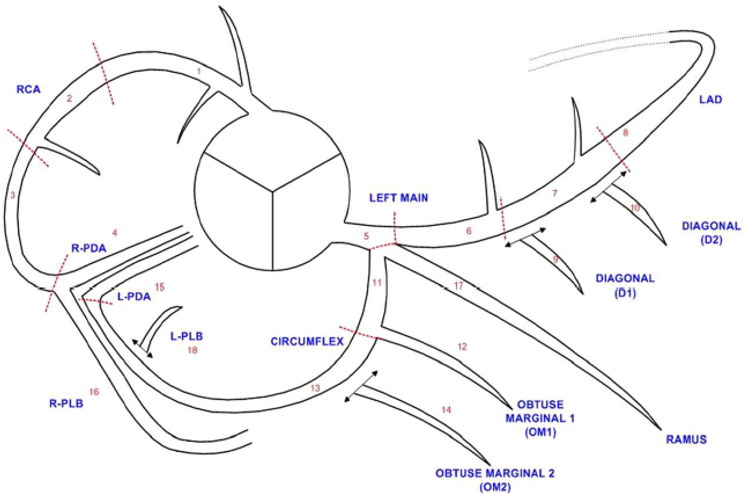
**PDA (R- 4 or L- 15), D (9, 10), OM (12, 14), PLB (R- 16, or L- 18), RI (17)

* Proximal (1) from aorta to one-half the distance to the acute margin of the heart, mid (2) from the end of proximal (1) to the acute margin of the heart, distal (3) from the acute margin of the heart to the origin of the R-PDA

** Left main (5) from the origin up to the bifurcation

*** LAD is subdivided into three segments of equal length: proximal (6), mid (7), and distal (8).

**** CX is subdivided into two segments of equal length: proximal (11) and distal (13).

According to SCCT, adapted from 1975 AHA model [1].

**CAD-RADS 2.0 grading system**

|  | **Stenosis grading** | **Interpretation CAD** |
| --- | --- | --- |
| **CAD-RADS 0** | 0% | No CAD |
| **CAD-RADS 1** | 1-24% | Wall irregularities |
| **CAD-RADS 2** | 25-49% | Mild stenosis |
| **CAD-RADS 3** | 50-69% | Intermediate stenosis |
| **CAD-RADS 4** | A: 70-99%  B: Left main ≥50% or 3 vessel ≥70% | Severe stenosis |
| **CAD-RADS 5** | 100% | Occlusion (CTO) |
| **CAD-RADS N*** | Non-diagnostic segment(s) >1,5 mm | CAD not ruled-out in specific segment |

***** CAD-RADS N grading system modified to CLEAR-CAD protocol.

**Plaque burden score**

|  | **Plaque burden** | **CAC** | **SIS*** | **Visual*** |
| --- | --- | --- | --- | --- |
| P1 | Mild | ≤100 | ≤2 | 1-2 vessels with mild plaque |
| P2 | Moderate / Intermediate | 101-300 | 3-4 | 1 -2 vessels with moderate / intermediate plaque or 3 vessels with mild plaque |
| P3 | Extensive | 301-999 | 5-7 | 3 vessels with moderate / intermediate plaque or 1 vessel with extensive plaque |
| P4 | Very extensive | >1000 | ≥8 | 2-3 vessels with very extensive plaque |

SIS = Segment involvement score

**HRP** (high-risk plaque)

- Positive remodeling, low-attenuation plaque (<30 HU), spotty calcification, napkin-ring sign

**I** (ischemia) not applicable in CLEAR-CAD

- + = CT-fractional flow reserve (CT-FFR) < 0.75 or CT-perfusion (CTP) with perfusion defect
- +/- = borderline CT-FFR (0.76-0.8) or CTP findings
- = CT-FFR >0.8 or CTP without perfusion defect

**S** (stent) not applicable in CLEAR-CAD

**G** (graft) not applicable in CLEAR-CAD

**E** (exceptions)

- Dissection, anomaly, (pseudo) aneurysm, vasculitis, external compression, other

Comments field

- Additions that cannot be described via the above
- Incidental findings

**CAD-RADS 2.0 grading system, CLEAR-CAD adjusted patient management for “N”**

**N** (non-diagnostic): If there is an inability to assess at least one segment, the modifier N will be applied. Always consider the reason for the non-diagnostic result and evaluate if obtaining a new scan would be beneficial. If needed, consult the CLEAR-CAD study team.

- If the CAD-RADS score is <3 in all diagnostic segments (>1.5 mm), including the LM and proximal LAD, than only the non-diagnostic segments are scored non-diagnostic and the modifier N is added to the overall CAD-RADS score. Additional imaging and treatment is according to the CLEAR-CAD protocol. In case of persistent symptoms suspected of obstructive CAD, additional imaging and treatment is according to the CLEAR-CAD protocol (CAD-RADS ≥3).
- If the CAD-RADS score is ≥3 in at least one segment (>1.5 mm), the overall CAD-RADS score is determined by the highest scoring segment. The non-diagnostic segments will be classified as modifier N. Treatment and additional imaging is according to the CLEAR-CAD protocol (CAD-RADS ≥3).
- If the LM or proximal LAD are considered of non-diagnostic image quality the overall CAD-RADS score at patient level is non-diagnostic, CAD-RADS N.

**CTCA run-in phase**

**Baseline protocols and reporting (before startup phase)**

Per centre local routine clinical care protocols are provided to the core lab

- Scan protocol
- Scanner (>64 slice CT scanner required)
- Sequence, kV, ref mAs
- Reconstruction (FOV, slice, gap, filter, FBP/ Iterative reconstruction (IR))
- Post-processing tool (functionalities, multiplanar reformation (MPR), curved-MPR, etc.)
- Contrast protocol
- Contrast medium (brand, generic and concentration) and power injector
- IDR (adjustment for kilovoltage (kV), body weight or other adjustment)
- Adjustment of injection rate, contrast dilution or contrast medium?
- Test-bolus or bolus tracking? Delay and threshold settings?

**CLEAR-CAD startup phase**

Workflow implementation of CLEAR-CAD step by step CTCA approach

- Patient preparation, scan protocol and contrast protocol, reconstructions, post-processing
- Reporting (CAD-RADS)
- CRF requirements, data anonymization and upload

CTCA scans acquired after implementation of the CLEAR-CAD step by step CTCA approach are provided to the core lab

- Baseline: 3 CTCA scans based on local routine clinical protocols
- Round 1: 3 CTCA scans after initial implementation of the CLEAR-CAD step by step CTCA approach
- Round 3: If image quality does not meet requirements for image quality and reporting criteria an additional 3 CTCA scans will be provided after feedback and adjustments
- Quality criteria

Scan

- Scan range
- Recommended target luminal attenuation (>325 HU aorta ascendens / proximal coronary artery)
- Diagnostic quality, no severe (motion) artifacts hampering interpretation
- Reconstruction and post-processing parameters
- FOV
- Slice thicknes
- Filters/kernels, FBP & IR
- Curved MPR
- Additional data (medication -, radiation dose – and contrast dose reports, including test-bolus scan and scan delay parameters and ECG data during CTCA acquisition)

Report

- Scan protocol
- Image quality
- CAD-RADS interpretation, including modifiers

**Hands-on workshop**

- Alignment with SKMS CTCA protocol recommendations
- Patient journey for CTCA with emphasis on CLEAR-CAD step by step CTCA approach
- Case readings and discussion (over 50 prepared cases), focus on CAD-RADS ≥3, modifiers and artifacts

**E-learning cases**

- Cases for self-study e-learning (n = 30)
- CAD-RADS 0-5 (>3 with invasive coronary angiography correlation and outcome)
- Artifacts

**On-site implementation support**

- Local support for implementation and execution of protocols during run-in phase, start-up phase and inclusion
- Data management support

1. **Non-Invasive functional imaging modalities: SOP and protocol**

**Background**

The purpose of this manual is to standardize the Cardiac Perfusion Imaging procedures(consisting of CMR(part 1) and PET/SPECT(part 2)) and among the clinical sites participating in the CLEAR-CAD trial. It is designed to be a practical guide to performing the procedures designated in the trial protocol.

In order to reduce variability in the Cardiac Perfusion studies among the clinical study sites, we ask that you follow the scanning procedures as outlined in this manual. The procedures are not difficult to complete, however, there are several important details that, if not implemented correctly, can have a significant effect on the diagnostic evaluation.

**Imaging Core Laboratory**

The Imaging Core Laboratory is located in the Amsterdam UMC. The role of the Imaging Core Laboratory for the CLEAR-CAD trial is to provide a quality assessment reading of each perfusion study scan submitted.

- 1. **Myocardial Perfusion Imaging using CMR**

**MRI Safety Guidelines**

Each clinical site should have written safety guidelines, practices and policies previously established for patients undergoing cardiac MRI as well as for the personnel participating in patient care in the MRI department. All study patients should undergo the routine screening process for the presence of contraindications to MRI that is routinely used by your department. Additional information about biomedical devices that may impose a risk to patients undergoing MRI is available on the internet at www.MRIsafety.com. Patients with implanted biomedical devices that are not MRI safe or that are MRI compatible but may result in imaging artifacts such as pacemakers, ICDs or CRT(Ds) are to be excluded. In addition, patients who have a history of severe claustrophobia or who are unable to lie flat, hold their breath for at least 10 seconds or who are too large to fit inside the bore of the scanner are to be excluded. Finally, absolute contraindications for adenosine stress testing include: i) patients with asthma and ongoing wheezing or history of steroid use or respiratory failure; ii) second- or third-degree AV block or sick sinus syndrome; iii) systolic blood pressure less than 90mm Hg; iv) recent use of dipyridamole or dipyridamole-containing medications; v) Methylxanthines (i.e. aminophylline caffeine or theobromine) should be held for at least 24 hours prior to the test.

**Patient Monitoring & Intravenous Access**

The study imaging procedures require that cardiac gating be used during image acquisition and ECG electrodes are to be placed on the patient’s chest. The administration of a gadolinium-based contrast agent and pharmacological stress agent during the CMR study requires that 2 peripheral IV accesses be established prior to the patient entering the scanner. One IV access needs to be sufficiently large for injection rates of 4.5 mL/s (typically 20 gauge) and placed in mid forearm or higher. In addition, connect the patient to an automated blood pressure monitor, to read the blood pressure prior to infusion (rest) of the pharmacological stress agent, and 1 minutes after the start of infusion (stress) and enter these measurements in the eCRF.

**Synopsis of CMR Stress Imaging Protocol**

**Step 1:** localizer and scout images

**Step 2:** SSFP cine imaging for 4-, 3- and 2-chamber views

**Step 3:** Test run perfusion sequence with 3 short axis slices *(see text for slice selection)*

- Native T1 map of the 3 short axis slices
- Measure blood pressure
- Start adenosine 140mcg/kg/min
- Measure blood pressure at 2 min
  - *(if no HR/symptom response augment adenosine dose to 210mcg/kg/min)*
- Start perfusion sequence at 4 min and inject stress contrast dose
  - (breath holding on arrival of contrast in RV cavity)

**Step 4:** Single shot LGE images (TI 350ms) short axis coverage

**Step 5:** SSFP cine imaging for short axis slices

**Step 6** TI scout in mid short axis slice followed by PSIR LGE of 3 long and all short axis slices

**Step 7** start rest perfusion sequence and inject rest contrast dose

**Detailed Imaging Protocol**

- **Step 1 - Localizer and scout images**

Obtain initial thoracic localizing images in sagittal, coronal, and axial planes ensuring correct cardiac positioning with the heart at the magnet bore center point. Using the localizer images, obtain scout images in mild expiration.

**2-chamber scout** use the axial scouts to prescribe a plane that bisects the LV parallel to the septum and through the apex.

*Axial localizer 2-chamber scout*


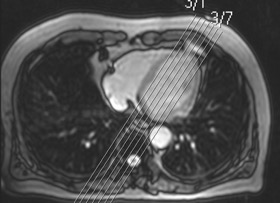

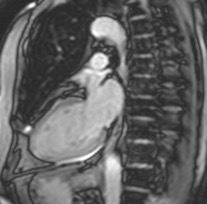


**Short axis scout** using the 2-chamber scout, prescribe a set of axial scouts perpendicular to the septum and the 2-chamber scout

*2-chamber scout short axis scout*


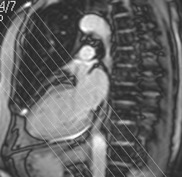

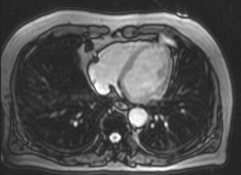

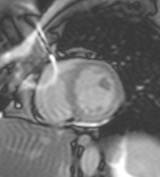


- **Step 2 - SSFP cine imaging long axis views**

**4-chamber cine** using the short axis scouts, prescribe a perpendicular plane through the middle of the LV cavity and the inferolateral wall of the RV (acute margin), and through the apex on the 2-chamber scout

*Short axis scout 2-chamber scout 4-chamber cine*


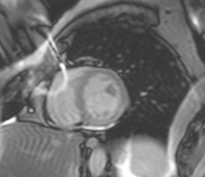

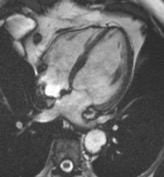

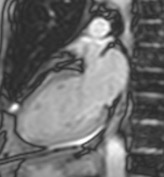


**3-chamber cine** using the short axis scout with LVOT view, prescribe a perpendicular plane through the middle of the LVOT and LV cavity, passing through the true 4-chamber apex

*Short axis scout 4-chamber cine 3-chamber cine*


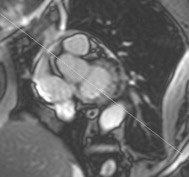

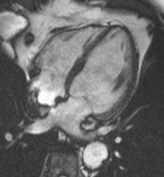

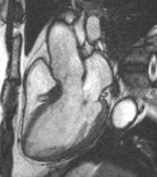


**2-chamber cine** using the mid short axis scout, prescribe a perpendicular plane through the middle of the LV, at an angle through the anterior and inferior wall, passing through the 4-chamber apex

*Short axis scout 4-chamber cine 3-chamber cine*


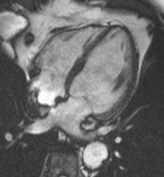

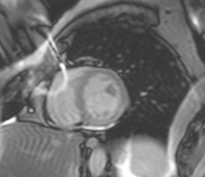

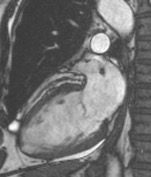


- **Step 3 - Stress perfusion imaging**


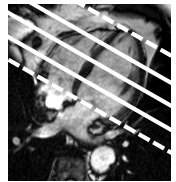
Blood pressure will be measured before the start of the stress medication (e.g., while obtaining the first pass perfusion test images). Stress first pass perfusion imaging is started after 4 minutes of continuous infusion of the pharmacological stress agent adenosine at a dosage described in Appendix II. See separate instructions if regadenoson is used. Set the contrast dose according to the contrast dosage in Appendix II, followed by 20mL saline infusion for flushing. Note that stress and rest dose is different. Both contrast and saline are injected at a rate of 4.5mL/s.

Prescribe 5 slices in short axis orientation equally divided over the LV, exclude the outer slices (dashed lines), and **acquire** the **three** inner **slices** (*basal, mid and apical*) covering all coronary territories. Check on the 3-chamber cine images to exclude the LVOT from the basal slice.

Before starting the pharmacological stress agent, native T1 maps are acquired on the three short axis slices (*basal, mid and apical*). These native T1 map images are repeated during stress, at 3 minutes of infusion of the pharmacological stress agent. Following the T1 maps, the contrast agent is injected during normal breathing, and when the contrast is about to enter the RV cavity the patient is asked to stop breathing in expiration, after which the contrast passes through the myocardium. Resume normal breathing and stop adenosine (for regadenoson: administer aminophylline) after the first-pass of contrast through the LV myocardium.


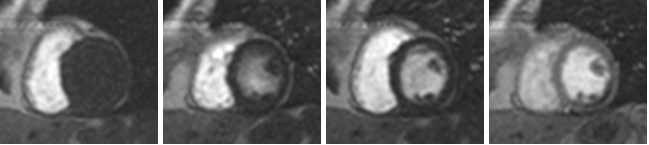


***stop breathing
in exspiration***

- **Step 4 – Single shot Late Gadolinium Enhancement with fixed TI**

After completion of the stress protocol, starting at approximately 1-2 minutes after contrast administration, prescribe a set of short axis slices perpendicular to the 4- and 2-chamber cines starting from above the AV valves and through the ventricles into the apex in a single non-breath-hold sequence acquisition with a fixed TI (350ms).

- **Step 5 - SSFP cine imaging short axis views**

Prescribe the set of cine short axis slices perpendicular to the 4- and 2-chamber cines, and covering the whole LV. Verify that the short axis images are perpendicular to the imaginary line from mid mitral valve to the apex. Set the flip angle slightly higher than for the long axis cines. Try to acquire the stack of short axis cines within 10 minutes.

*4-chamber cine 2-chamber cine short axis view*


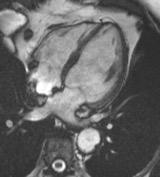

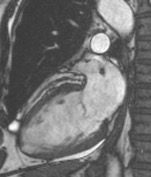

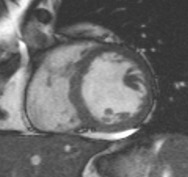


- **Step 6 - Late Gadolinium Enhancement**

Use a TI scout/look-locker/cine IR in short axis orientation in order to set the correct inversion time (TI) and null the signal of normal/viable myocardium on the late gadolinium enhancement (LGE) images. It is preferred to set the TI slightly longer. Acquire 2D segmented LGE images of all long axis and short axis slices, and identical to the cine locations.


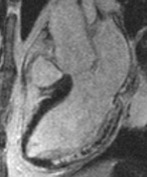

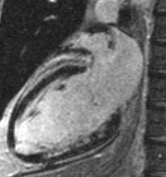

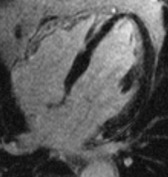

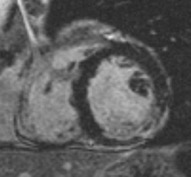


- **Step 7 - Rest perfusion imaging**

Set the contrast infusion dose according to the contrast dosage in Appendix II at a rate of 4.5mL/sec and set the saline infusion of 20mL for flushing.

Use the identical number and location of slices as during the stress perfusion acquisition. During normal breathing the contrast agent is injected, and when the contrast is about to enter the RV cavity the patient is asked to stop breathing in expiration, after which the contrast passes through the myocardium.

Please check all images and verify that no short axis cine or LGE slice is missing before the patient leaves the scan table.

**Recommended sequence parameters**

| **Cine imaging** | |
| --- | --- |
| pulse sequence | 2D steady-state free precession with retrospective ECG gating   *(True FISP, balanced FFE, FIESTA)* |
| matrix | 256 (base resolution) |
| spatial resolution | ~ 1.5 x 2.0 mm |
| temporal resolution | < 40 ms |
| flip angle | 60^O^ at 1.5T / 45 ^O^ at 3T |
| parallel imaging factor | ≤ 2 |
| slice thickness | 5 mm |
| slice gap | 5 mm / 100% |

| **First pass perfusion imaging** | |
| --- | --- |
| pulse sequence | 2D saturation recovery, spoiled gradient echo  *(TurboFLASH, T_1_-weigthed TFE, SPGR)* ***please*** ***NO SSFP*** |
| matrix | 192 at 1.5T / 256 at 3T (base resolution) |
| spatial resolution | ~ 1.9 x 2.4 mm at 1.5T / ~ 1.4 x 1.8 mm at 3T |
| saturation time* | 130 ms (maximized for heart rate in **1 RR** interval) |
| flip angle | 15^O^ at 1.5T / 12 ^O^ at 3T |
| slice thickness | 8 mm |
| slice gap | 2 mm / 25% |
| number of slices per heart beat | 3, equally divided over the heart, excluding the LVOT |
| temporal resolution | 120 ms |
| parallel imaging factor | 2 at 1.5T / 3 at 3T |
| scan duration | 70 heart beats |

| **Single shot Late Gadolinium Enhancement** | |
| --- | --- |
| pulse sequence | 2D, inversion recovery, steady-state free precession |
| matrix | 208 at 1.5T / 256 at 3T (base resolution) |
| spatial resolution | ~ 1.5 x 2.3 mm |
| ECG trigger | every other heartbeat, trigger delay to image in mid diastole |
| Inversion pulse | non-selective |
| inversion time (TI) | **350 ms** |
| flip angle | 25^O^ at 1.5T / 20 ^O^ at 3T |
| parallel imaging factor | 2 at 1.5T / 3 at 3T |
| slice thickness | 8 mm |
| slice gap | 2 mm / 25% |

| **Late Gadolinium Enhancement** | |
| --- | --- |
| **pulse sequence** | 2D, inversion recovery, spoiled gradient echo, with PSIR  *(FLASH, T_1_-weighted TFE, SPGR)* |
| **matrix** | 256 (base resolution) |
| **spatial resolution** | ~ 1.5 x 2.0 mm |
| **ECG trigger** | every other heartbeat, trigger delay to image in mid diastole |
| **Inversion pulse** | non-selective |
| **inversion time (TI)** | optimize to null signal of normal myocardium |
| **flip angle** | 25^O^ at 1.5T / 20 ^O^ at 3T |
| **parallel imaging factor** | ≤ 2 |
| **slice thickness** | 5 mm |
| **slice gap** | 5 mm / 100% |
| **band width** | 140 Hz/pixel at 1.5T / 350 Hz/pixel at 3.0T |

**Contrast agent dosage recommendations**

During stress and rest perfusion imaging contrast dosage is slightly higher (0.12mmol) than the rest (0.08mmol) perfusion contrast dosage. When rest perfusion is not performed, the rest contrast dose may be injected shortly after stress perfusion. It is also allowed to use 0.10mmol of the contrast agent per acquisition, if image quality for visual assessment is sufficient.
The table below displays the volume of contrast for each acquisition (stress or rest), per patient weight. Contrast dose used is equal at 1.5T and 3T.

| **ProHance® - gadoteridol 0.5mmol/mL** | | | | | | | | | | | | | |
| --- | --- | --- | --- | --- | --- | --- | --- | --- | --- | --- | --- | --- | --- |
| weight (kg) | 50 | 55 | 60 | 65 | 70 | 75 | 80 | 85 | 90 | 95 | 100 | 105 | 110 |
| stress (ml) | 13 | 14 | 15 | 16 | 18 | 19 | 20 | 21 | 22 | 24 | 26 | 26 | 28 |
| rest (ml) | 7 | 8 | 9 | 10 | 11 | 11 | 12 | 12 | 13 | 14 | 14 | 16 | 16 |
| **Dotarem® - gadoterate meglumine 0.5mmol/ml** | | | | | | | | | | | | | |
| weight (kg) | 50 | 55 | 60 | 65 | 70 | 75 | 80 | 85 | 90 | 95 | 100 | 105 | 110 |
| stress (ml) | 13 | 14 | 15 | 16 | 18 | 19 | 20 | 21 | 22 | 24 | 26 | 26 | 28 |
| rest (ml) | 7 | 8 | 9 | 10 | 11 | 11 | 12 | 12 | 13 | 14 | 14 | 16 | 16 |
| **Clariscan® - gadoterate meglumine 0.5mmol/ml** | | | | | | | | | | | | | |
| weight (kg) | 50 | 55 | 60 | 65 | 70 | 75 | 80 | 85 | 90 | 95 | 100 | 105 | 110 |
| stress (ml) | 13 | 14 | 15 | 16 | 18 | 19 | 20 | 21 | 22 | 24 | 26 | 26 | 28 |
| rest (ml) | 7 | 8 | 9 | 10 | 11 | 11 | 12 | 12 | 13 | 14 | 14 | 16 | 16 |
| **Gadovist® - gadobutrol 1.0mmol/ml** | | | | | | | | | | | | | |
| weight (kg) | 50 | 55 | 60 | 65 | 70 | 75 | 80 | 85 | 90 | 95 | 100 | 105 | 110 |
| stress (ml) | 6 | 7 | 8 | 8 | 9 | 9 | 10 | 10 | 11 | 12 | 12 | 13 | 14 |
| rest (ml) | 4 | 4 | 4 | 5 | 5 | 6 | 6 | 7 | 7 | 7 | 8 | 8 | 8 |

**Pharmacological stress agent and dosage recommendations**

Patients should be instructed to avoid consumption of any products containing methylxanthines, including caffeinated coffee, tea or other caffeinated beverages, caffeine-containing drug products, aminophylline and theophylline for at least 12 hours before a scheduled CMR scan.

The dosage for **regadenoson** is **0.4mg/5mL** in a **single dose**, and should be administered in 10 seconds into an intravenous catheter, with 5mL saline flush immediately afterwards. The stress perfusion sequence is started approximately 1 minute after injection of regadenoson. After the acquisition of the stress perfusion images, the effects of regadenoson should be reversed with 50 mg aminophylline intravenously.

The dosage for **adenosine** is **140mcg/kg/min** for 2 minutes at which blood pressure and heart rate are measured and in case the patient has no symptoms nor increase in heart rate, the adenosine dose is augmented to **210mcg/kg/min**. Contrast injection and stress imaging starts at 4 minutes after starting adenosine. In the table the different pump settings for administration in relation to body weight are displayed.

| **adenosine dosage 3mg/mL** | | | | | | | | | | | | | | |
| --- | --- | --- | --- | --- | --- | --- | --- | --- | --- | --- | --- | --- | --- | --- |
| weight (kg) | | 50 | 55 | 60 | 65 | 70 | 75 | 80 | 85 | 90 | 95 | 100 | 105 | 110 |
| **140mcg** | mg/min | 7.0 | 7.7 | 8.4 | 9.1 | 9.8 | 10.5 | 11.2 | 11.9 | 12.6 | 13.3 | 14.0 | 14.7 | 15.4 |
|  | mL/min | 2.3 | 2.6 | 2.8 | 3.1 | 3.3 | 3.6 | 3.8 | 4.1 | 4.3 | 4.6 | 4.8 | 5.1 | 5.3 |
|  | mL/hr. | 140 | 154 | 168 | 182 | 196 | 210 | 224 | 238 | 252 | 266 | 280 | 294 | 308 |

| **210mcg** | mg/min | 10.5 | 11.6 | 12.6 | 13.7 | 14.7 | 15.8 | 16.8 | 17.9 | 18.9 | 20.0 | 21.0 | 22.1 | 23.1 |
| --- | --- | --- | --- | --- | --- | --- | --- | --- | --- | --- | --- | --- | --- | --- |
|  | mL/min | 3.5 | 3.9 | 4.2 | 4.6 | 5.0 | 5.4 | 5.7 | 6.1 | 6.5 | 6.9 | 7.2 | 7.6 | 8.0 |
|  | mL/hr. | 210 | 231 | 252 | 273 | 294 | 315 | 336 | 357 | 378 | 399 | 420 | 441 | 462 |

- 1. **Myocardial Perfusion Imaging using SPECT and PET**

**General considerations**

All patient preparation, stress tests and image acquisitions should be performed according to local protocol and adhere to the EANM 2015 updated procedural guidelines for myocardial perfusion imaging and the EANM 2020 procedural guidelines PET/CT for quantitative myocardial perfusion imaging.

For the purpose of the CLEAR-CAD study both stress and rest perfusion imaging are mandatory.

**Patient preparation**

In general heavy meals should be avoided before a stress test. Medications that may interfere with responses to a stress test (anti-anginal drugs, dipyridamole or dipyridamole containing medication) should be interrupted if possible, and the patient must abstain from caffeine containing drugs and beverages (see Table).

**Stress tests**

In case of SPECT imaging dynamic exercise is the first test of choice. However, patients must be able to exercise to a workload of at least 85% of age-adjusted maximal predicted heart rate (220-age). For PET imaging pharmacological stress is the way to go.

Dynamic exercise should not be performed in patients who cannot achieve an adequate hemodynamic response because of non-cardiac limitations including lung diseases, peripheral vascular disease, musculoskeletal diseases, neurological diseases or poor motivation. These patients should undergo pharmacological stress perfusion testing (Figure). There are two groups of medication that can be used for pharmacological stress: (1) the vasodilators adenosine, regadenoson, and dipyridamole and (2) the sympathomimetic agent dobutamine.

All stress procedures must be supervised by a qualified and appropriately trained health-care professional being either physician, nurse or technician. Non-medical staff (depending on national regulations) has to operate according to the locally approved procedure and may commonly work under the direct or indirect supervision of a physician. The staff must be experienced in the selection of the most appropriate form of stress for the clinical question being asked and must have the clinical skills to recognize patients with an increased risk of complications. Appropriate facilities for cardiopulmonary resuscitation must be available and the staff must have up-to-date knowledge of advanced life support (ALS) techniques or intermediate life support and immediate access to personnel with ALS expertise.

| ***Medication*** | ***Exercise*** | ***Vasodilator*** | ***Dobutamine (±atropine)*** |
| --- | --- | --- | --- |
| **Nitrates** | interrupt  (3-5 half-lives) | interrupt  (3-5 half-lives) | interrupt  (3-5 half-lives) |
| **Beta blockers** | interrupt  (3-5 half-lives) | interruption recommended  (3-5 half-lives) | interrupt  (3-5 half-lives) |
| **Calcium antagonists** | interrupt  (3-5 half-lives) | interruption recommended  (3-5 half-lives) | interrupt  (3-5 half-lives) |
| **Drugs, food, beverages with caffeine** | continue* | interrupt  (> 12 h prior to stress) | continue* |
| **Drugs, food, beverages with methylxanthines** | continue* | interrupt  (> 12 h prior to stress) | continue* |
| **Dipyridamole** | continue* | interrupt  (> 24 h prior to stress) | continue* |

* In order not to rule out any of the stress modalities it is recommended for every patient to interrupt caffeine-containing beverages (coffee, tea, cola etc.), foods (chocolate etc.) and caffeine-containing medication (some pain relievers, stimulants and weight-control drugs) as they antagonize the vasodilator effects.

***Selection of stress test modality for SPECT myocardial perfusion imaging***


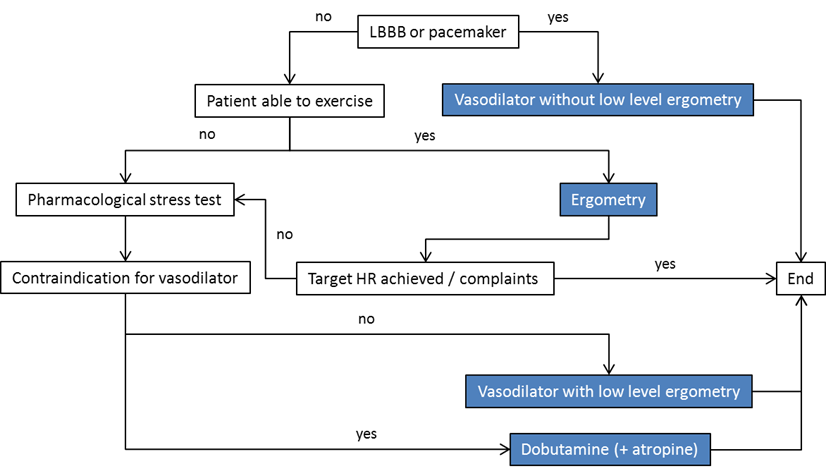


* The option dobutamine (+ atropine) should only be used when available and when available only with sufficient clinical experience. However, in clinical practice pharmacological stress with a vasodilator (i.e., adenosine or regadenoson) will suffice in most patients, expect for those patients with (severe) constrictive COPD.

**Tracers**

All commonly available myocardial perfusion tracers for PET and SPECT are allowed.

**Image acquisition**

Image acquisition depends on the modality (SPECT vs. PET) and tracers used. The imaging protocols should be performed according to local protocol and adhere to the EANM 2015 updated procedural guidelines for myocardial perfusion imaging and the EANM 2020 procedural guidelines PET/CT for quantitative myocardial perfusion imaging.

Again, for the purpose of the CLEAR-CAD study both stress and rest perfusion imaging are mandatory.

**References**

1. Leipsic J, Abbara S, Achenbach S, et al. SCCT guidelines for the interpretation and reporting of coronary CT angiography: a report of the Society of Cardiovascular Computed Tomography Guidelines Committee. J Cardiovasc Comput Tomogr. 2014;8(5):342-58.
